# EFFECTIVENESS OF REHABILITATION INTERVENTIONS IN PATIENTS WITH COLORECTAL CANCER: AN OVERVIEW OF SYSTEMATIC REVIEWS

**DOI:** 10.2340/jrm.v57.40021

**Published:** 2025-01-24

**Authors:** Mengzhe YANG, Bhasker AMATYA, Sana MALIK, Krystal SONG, Stefanie MARCELLA, Catherine VOUTIER, Fary KHAN

**Affiliations:** 1 Department of Rehabilitation Medicine, Royal Melbourne Hospital, Parkville, Victoria; 2 Department of Medicine (Royal Melbourne Hospital), University of Melbourne, Parkville, Victoria; 3 Australian Rehabilitation Research Centre, Royal Melbourne Hospital, Parkville, Victoria; 4 Department of Rehabilitation, Peter MacCallum Cancer Centre, Parkville, Victoria, Australia; 5 Albany Medical College, Albany, New York, USA

**Keywords:** rehabilitation, colorectal neoplasms, colorectal cancer, systematic review, critical appraisal

## Abstract

**Objective:**

To evaluate existing evidence from published systematic reviews for the effectiveness and safety of rehabilitation interventions in adult patients with colorectal cancer.

**Methods:**

A comprehensive literature search was conducted using medical/health science databases up to October 2024. Bibliographies of pertinent articles, journals, and grey literature were searched. Three reviewers independently selected potential reviews, assessed methodological quality, and graded the quality of evidence for outcomes using validated tools.

**Results:**

Sixty systematic reviews (761 randomized controlled trials) evaluated 5 categories of rehabilitation interventions. Over half of the included reviews (*n* = 31) were of moderate–high quality. The findings suggest: moderate-quality evidence for exercise interventions for improving physical fitness and quality of life; high-quality evidence for nutritional interventions in reducing postoperative infections; high-quality evidence for multimodal prehabilitation for improved preoperative functional capacity; moderate-quality evidence for nutritional interventions for improving humoral immunity, reducing inflammation, and length of stay; moderate-quality evidence for acupuncture in improving gastrointestinal functional recovery; psychosocial interventions in improving short-term quality of life and mental health, and lifestyle interventions for improved quality of life.

**Conclusion:**

Rehabilitation interventions yielded positive effects across multiple outcomes. However, high-quality evidence is still needed to determine the most effective rehabilitation approaches for patients with colorectal cancer.

## LAY ABSTRACT

Colorectal cancer and its treatments can cause significant disability and morbidity. A range of rehabilitation interventions is currently studied in patients with colorectal cancer. This overview systematically evaluated evidence from published systematic reviews of randomized clinical trials to determine the effectiveness of rehabilitation interventions to improve clinical outcomes in patients with colorectal cancer. The findings suggest moderate-quality evidence for exercise interventions for improving physical fitness and quality of life; high-quality evidence for nutritional interventions in reducing postoperative overall infectious complication rates; high-quality evidence for multimodal prehabilitation for improved preoperative functional capacity; moderate-quality evidence for nutritional interventions for improved immunity, reduced inflammation, and hospital length of stay. There was also moderate-quality evidence for acupuncture interventions in improving gastrointestinal functional recovery, psychosocial interventions in improving short-term quality of life and mental health, and lifestyle interventions for improved quality of life. Overall, rehabilitation interventions have positive effects across multiple outcomes for patients with colorectal cancer, but high-quality evidence is still lacking.

Colorectal cancer (CRC) is the third most commonly diagnosed cancer in males and the second in females (1). Over 1.9 million new CRC cases and 930, 000 deaths were estimated worldwide in 2020, and the global burden of CRC is projected to increase to 3.2 million new cases and 1.6 million deaths by 2040 (2). The mainstay of CRC treatment includes surgical resection and chemoradiotherapy depending on the tumour staging. Colorectal cancer and its treatments can have a multidimensional impact on an individual’s functional capacity and societal participation due to various issues such as irregular bowel movements, weight loss, fatigue, pain, and insomnia (3). Further, colorectal surgery is associated with a high risk of postoperative complications and mortality (4) and chemotherapy can lead to a range of toxicities including mucositis, emesis, diarrhoea, febrile neutropenia, fatigue, hair loss, hand–foot syndrome, peripheral neuropathy, and cardiotoxicity (5). Many CRC patients experience reduced cardiorespiratory fitness and cancer-related fatigue (CRF) during and following treatment (6, 7). There is also a high prevalence of depression (up to 57%) and anxiety (up to 42%) in patients with CRC (PwCRC) (8). These physical and psychological consequences limit activities of daily living and restrict societal participation many years after the diagnosis (9). These complex and multifaceted issues highlight the importance of an integrated multidisciplinary approach, including rehabilitation.

Rehabilitation plays an integral role within the continuum of care for PwCRC and aims to maximize an individual’s functional independence, participation, psychological well-being, and quality of life (QoL). Rehabilitation programmes can be uni-disciplinary or coordinated interdisciplinary care that is individualized and goal-oriented for PwCRC at any stage of their cancer treatment based on their needs. CRC treatments are traditionally limited to patients with good physical reserve and functional independence. For patients with more significant frailty, prehabilitation can improve their functional capacity and potentially broaden their treatment options (10, 11). In those undergoing CRC surgery or adjuvant treatment, rehabilitation interventions such as nutritional interventions and exercise therapy serve important roles in boosting immunity, reducing treatment-related complications, improving hospital length of stay (LOS), and optimizing functional capacity (12–15). Further, rehabilitation interventions involving exercise, and cognitive and psychosocial interventions, have positive effects towards improved physical fitness, reduced CRF, improved functional independence, QoL, and psychological wellbeing in cancer survivors (16–20). Though various systematic reviews have evaluated the effects of rehabilitation interventions in PwCRC, these published reviews vary in scope, methodology, and quality, with diverse and sometimes discordant conclusions. The specific type, mode of delivery, and duration of interventions within primary studies of these published systematic reviews were often significantly heterogeneous. As a result, rehabilitation-specific evidence-based guidelines and recommendations for the most effective rehabilitation interventions remain limited in this cohort (21–23).

To our knowledge, systematic reviews of the effectiveness of comprehensive rehabilitation strategies for CRC have not been appraised to date. Therefore, this review aimed to systematically evaluate and summarize the evidence from the published systematic reviews regarding the effectiveness and safety of rehabilitation interventions towards improving physical impairments, functional outcomes, and participation in adult PwCRC (Box 1). We envisage that this overview of systematic reviews will provide useful information to guide clinical practice in formulating rehabilitation programmes and identify gaps in current knowledge in this cohort.

Box 1. Research questions:

- Are rehabilitation interventions effective in improving physical fitness and reducing cancer- or treatment-related symptoms (e.g., gastrointestinal symptoms, CRF, incontinence etc.)?
- Are rehabilitation interventions effective in reducing post-treatment complications rates, shortening hospital LOS, improving patient’s functional independence?
- Are rehabilitation interventions effective in improving patients’ psychological well-being, societal participation, QoL, and overall survival?

## MATERIALS AND METHODS

### Study design

This is an overview of published systematic reviews and followed the Preferred Reporting Items for Systematic Reviews and Meta-Analysis report specification (24). The protocol is available in the PROSPERO registration (ID: CRD42023427886).

### Inclusion and exclusion criteria

Systematic reviews and/or meta-analyses that investigated the effect of rehabilitation interventions conducted in any setting (inpatient, home-based, ambulatory settings) in adult patients (> 18 years) with CRC were included. Studies published between 2013 and 2024 and limited to English language only were included. Systematic reviews that involved other cancer groups but provided CRC sub-group data were also included. Due to the large number of systematic reviews identified during the initial screening phase, an additional inclusion criterion was added after the submission of the study protocol to select reviews that included only randomized controlled trials (RCT) as their primary studies.

The exclusion criteria included: reviews focused solely on diagnostic, pharmacological, and/or surgical interventions; reviews that included other study types such as observational studies or those conducted in paediatric populations; reviews that assessed the feasibility of rehabilitation programme implementation, and behavioural interventions aimed to promote exercise and lifestyle behaviours; narrative reviews; theses; health technology appraisal; and reviews listed only in conference proceedings.

### Types of outcome measures

The WHO International Classification of Functioning, Disability and Health (ICF) model (25) was used to conceptualize the outcomes in this review.

The primary outcomes included:

- Improvement in CRC and/or cancer treatment-related physical impairment/symptoms and physical fitness status.
- Improvement in functional outcomes.
- Improvement in participation outcome quantified by QoL and psychosocial gains.

Secondary outcomes included:

- Health service utilization (e.g., hospital LOS, readmission rates).
- Mortality rates, cost-effectiveness, and adverse events.

### Search methods

We searched the following databases to identify relevant studies: Embase, Emcare, Medline, and PsycINFO, Cochrane Library, and the Cumulative Index of Nursing and Allied Health (CINAHL). The search strategy was adapted from another rehabilitation systematic review (26) and run in all databases initially in June 2023 and an updated search was performed on 30 October 2024. The systematic review/meta-analysis filter from the BMJ Best Practice EBM Toolkit was used. A grey literature search was conducted using Google Scholar, Open Access Theses and Dissertations (OATD), National Technical Information Service (NTIS), WHO’s International Repository for Information Sharing (IRIS), and the INAHTA HTA Database. The CitationChaser program (https://estech.shinyapps.io/citationchaser/) was used to extract all citing and cited papers from the included studies list. Reference checking of relevant articles and journals located no additional reviews. EndNote x9 (Clarivate; https://clarivate.com/), was used to download all database results, and retrieve full text, which was uploaded into Covidence (https://www.covidence.org/), which auto-removed duplicate records. Records that were duplicates but not marked by Covidence as such were indicated manually.

A full description of the search strategy is listed in Appendix SI.

### Study selection and data extraction

Two authors (MY, CV) independently screened and shortlisted all abstracts and titles to extract relevant systematic reviews based on the selection criteria. Any disagreements were resolved by consensus discussion with a third author (BA). All relevant data were extracted by 3 authors (MY, SD, SM) and included: publication and search date; characteristics of included primary studies and study subjects; interventions and comparisons; findings/outcomes including pooled results from meta-analysis; and limitations. Any discrepancies were resolved by group discussions and by re-reviewing the study.

### Quality of evidence

Five authors (MY, KS, SM, SD, BA) assessed the methodological quality of included studies, using the revised “A MeaSurement Tool to Assess systematic Reviews” appraisal tool (27). The AMSTAR-2 (https://amstar.ca/) includes 16 assessment items, 7 of which are considered critical methodological items for high-quality studies. For this overview, item 7 (Did the review authors provide a list of excluded studies and justify the exclusions?) was re-categorized as a non-critical methodological item and not deemed an essential requirement for systematic reviews, as most included studies did not include a list of excluded studies. Any disagreements were resolved through final group consensus. The quality of the primary studies within the systematic reviews was not reassessed.

The Grade of Recommendation, Assessment, Development and Evaluation (GRADE) tool (https://www.gradeworkinggroup.org/) (28) was used to assess the quality of evidence for each outcome. The quality of evidence was rated as (28): “High-quality”: very confident that the true effect lies close to that of the estimate of the effect; “Moderate-quality”: moderately confident in the effect estimate, such that the true effect is likely to be close to the estimate of the effect, but there is a possibility that it is substantially different; “Low-quality”: confidence in the effect estimate is limited, and the true effect may be substantially different from the estimate of the effect; “Very low-quality”: very little confidence in the effect estimates and the true effect is likely to be substantially different from the estimate of the effect.

Any discrepancies were resolved by final consensus amongst the reviewers.

## RESULTS

**Fig. 1:**
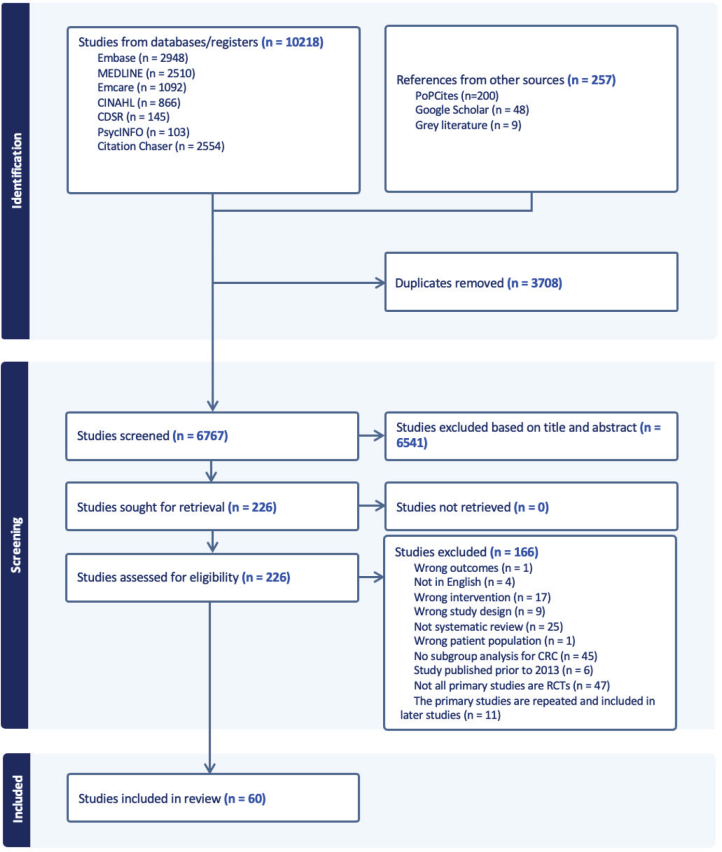
Preferred Reporting Items for Systematic reviews and Meta-Analyses (PRISMA) flow diagram showing a selection of review.

The search retrieved 10,218 articles. Of these, 226 articles met the title/abstract inclusion criteria and were selected for full text review. Overall, 60 systematic reviews and/or meta-analyses (12–20, 29–79) including 2 reviews published in the Cochrane Library database (18, 67) were included. A Preferred Reporting Items for Systematic Reviews and Meta-Analyses (PRISMA) flow diagram of the study selection process is shown in Fig. 1. The included reviews were published between 2013 and 2024, consisting of 761 RCTs, with more than 65,841 participants with CRC. The majority of reviews focused solely on CRC, with 4 reviews including other cancer types with subgroup analysis data on PwCRC (13, 14, 42, 68). Meta-analyses were performed in 53 studies, with the rest providing qualitative analyses of the findings (29, 39, 40, 50, 61, 62, 64). The characteristics of the included reviews are summarised in Table I.

**Table I:** Characteristics of included systematic reviews

Author and year	Outcome measure	Intervention	Included studies, search dates and meta-analysis, *n*	Participants	Main findings
**Exercise interventions**					
Da Silva Bezerra et al. (29) 2021	QoL: FACT-C, FACT-G, SF-12	Aerobic exercises (2 RCTs); semi-supervised aerobic and resisted exercises (1 RCT); personalised exercise (1 RCT) Comparator: no exercise or usual activities	4 RCTs Search date 2020 Meta-analysis: No	CRC survivors (*n* = 315)	No significant increase in QoL in CRC patients undergoing exercise vs control group except for 1 study
Cramer et al. (30) 2013	Primary outcomes: health-related QoL, fatigue, physical fitness Secondary outcomes: survival, tumour-associated biomarkers, safety	Exercise interventions with no treatment or with any active treatment Comparator: no treatment	5 RCTs Search date: 20 Dec 2012 Meta-analysis: Yes (3 RCTs)	PwCRC 3–24 months after cancer treatment (*n* = 238)	Primary outcomes: 1. Moderate intensity exercise showed no evidence for short-term effects on QoL (SMD = 0.18; 95% CI: −0.39, 0.76; *p* = 0.53) (3 RCT, *n* = 157) or fatigue (SMD = 0.18; 95% CI −0.22, 0.59; *p* = 0.38) (3RCTs, *n* = 157) 2. Strong evidence for short-term improvements of physical fitness after aerobic exercise (SMD = 0.59; 95% CI 0.25, 0.93; *p* < 0.01) (3RCTs, *n* = 152) Secondary outcomes: 1. Moderate intensity exercise showed more pro-inflammatory immune state (decreased antagonist/cytokine ratio) and increased DNA damage but not low-intensity exercise (2 RCTs) 2. Long term follow-up at 12 months, no differences regarding QoL or fatigue, but improved physical fitness in the exercise group (1 RCT) 3. No survival rates or safety data reported
Jung et al. (31) 2021	QoL, PA, fatigue, VO2 max, BMI	Home-based PA (aerobic exercise, and a combination of resistance exercises) +/-nutrition and supervision exercises for behaviour change Comparator: Usual care (educational materials or brief advice regarding maintenance of usual behaviour patterns) and no intervention	7 RCTs Search date: 4 Feb 2019 Meta-analysis: Yes	CRC survivors who completed primary treatment (*n* = 803)	1. PA intervention significantly improved disease-specific QoL vs usual care (MD, 3.74; 95% CI, 0.22–7.25, *p* = 0.04) (5 RCTs, *n* = 173) 2. Significant increase in PA for those receiving the intervention (SMD, 0.80; 95% CI, 0.28–1.32, *p* = 0.003) (3RCTs) 3. Significant improvement in VO2 max in the intervention group (MD, 3.19; 95% CI, 1.24– 5.13; *p* = 0.001) (2 RCTs, *n* = 257) 4. PA was not found to significantly decrease BMI vs usual care (MD, −0.21; 95% CI, −0.48 to 0.06; *p* = 0.12) (4 RCTs) 5. PA intervention did not show significant effect for reducing participants’ fatigue (SMD, 0.17; 95% CI, −0.37 to 0.72; *p* = 0.54) (4 RCTs, *n* = 127)
Gao et al. (16) 2020	Psychosocial. (QoL, fatigue, depression) Physical function (cardiopulmonary fitness, muscle strength, body flexibility Body composition (BMI, waist circumference, lean mass) Metabolic growth factors (insulin) Tumor-related biomarkers	Aerobic exercises, either alone or combined with resistance training, under supervision or at home Either alone or in combination with other interventions Comparator: usual care or usual lifestyle, or variable exercise intensity	20 RCTs Search date: May 2020 Meta-analysis: Yes (11 RCTs)	CRC survivors after primary cancer treatment (*n* = 1,223)	Psychosocial outcomes: - No significant increase in QoL (SMD = 0.22, 95% CI: –0.01, 0.45, *p* = 0.06) (6 RCTs, *n* = 315) - No significant improvement in fatigue (SMD = 0.08, 95% CI: –0.08, 0.24, *p* = 0.30) (6 RCTs, *n* = 628) - No significant effect for depression (SMD = –0.21, 95% CI: - 0.48,0.07, *p* = 0.14) (3 RCTs, *n* = 217) - No significant effect for anxiety (SMD = –0.34 95% CI: –0.68, 0.00, *p* = 0.05) (2 RCTs, *n* = 146) Physical outcomes: - Significantly increased VO2 peak (SMD = 0.72, 95% CI: 0.32 to 1.11, *p* = 0.0004) (3 RCTs, *n* = 107) - Insignificant outcomes for 6MWT, 30-s chair–stand test, push-up tests
					Body composition: - No significant improvement in BMI (SMD = –0.16 95% CI: –0.69, 0.37, *p* = 0.55) (6 RCTs, *n* = 701) - No significant improvement in body fat percentage (SMD = –0.11 95% CI: –0.35, 0.14, *p* = 0.39) (4RCTs, *n* = 262); only high-intensity aerobic training subgroup reduced the body fat percentage - No significant effect for waist circumference (SMD = –0.01 95% CI: –0.27,0.25, *p* = 0.93) (3 RCTs, *n* = 235) Metabolic growth factors: - Significant decrease in fasting insulin & insulin resistance (*p* = 0.0009 & *p* = 0.0002) Biomarkers: - Decreased levels sICAM-1in aerobic exercise group - Moderate-intensity exercise was associated with proinflammatory immune state and increased DNA damage
Dun et al. (32) 2020	CRF, QoL	Exercise or PA of moderate intensity Comparator: Usual care or no intervention or routine rehabilitation	10 RCTs Search date: April 2019 Meta-analysis: Yes (10 RCTs)	PwCRC who underwent elective colorectal surgery (*n* = 934)	1. Significantly reduced cancer-related fatigue (SMD = –1.34, 95% CI: –2.16, –0.53, *p* = 0.001) (9 RCT, *n* = 874) - Intervention time < 12 weeks (SMD = 3.20, 95% CI –5.75, –0.65) results in lower CRF than control group - No significant effects for intervention time > 12 weeks 2. Significantly improved social factors of QoL (SMD = 0.67 95% CI: 0.15, 1.19, *p* = 0.012) (3 RCTs, *n* = 138) - No significant difference in QoL cognitive effects (SMD = 0.51, *p* = 0.233) & physiological factors (SMD = 0.54, *p* = 0.097)
Geng et al. (33) 2023	Fatigue: FACIT-F, FACT-F, RFPS, BFI, MFI, FSI	Exercise/PA (aerobic exercise, resistance exercise, mixed mode, or other exercise) Comparator: Routine care group or blank control group	8 RCTs Search date: 1 April 2022 Meta-analysis: Yes	PwCRC regardless of pre-, during or following treatment (*n* = 542)	PA intervention significantly reduced CRF (SMD = –0.46; 95% CI –0.76, – 0.15, *p* = 0.003) (8 RCTs, *n* = 509) Subgroup based on intervention length: - Significant improvement when intervention length > 6 months (SMD = –0.54, 95% CI –0.81, –0.27, *p* = 0.0001) (4 RCTs, *n* = 221) - No difference when duration < 6 months Subgroup based on weekly duration: - Significant improvement when weekly duration of PA < 150 min/week (SMD = –0.67, 95% CI –1.15, –0.19, *p* = 0.006) - No effect on CRF if PA > 150 min/week (SMD = –0.12; 95% CI: –0.50, 0.26, p = 0.54)
Wang et al. (34) 2022	Primary outcome: health-related QoL Secondary outcomes: fatigue score, emotional functioning, anxiety, weight	Exercise interventions Comparator: usual care	5 RCTs Search date: Sept 2020 Meta-analysis: Yes	CRC survivors who can walk unaided (*n* = 159)	1. Significantly improved health-related QoL after exercise intervention (SMD = 2.79, 95% CI: 1.66, 3.92, *p* < 0.00001) 2. Significantly reduced fatigue score after exercise intervention (SMD = –2.21; 95% CI: –3.22 to –1.20, *p* < 0.0001) (3 RCTs) 3. No significant impact on emotional function, weight, or anxiety score (SMD = –0.93, 95% CI: –2.5, 0.64, *p* = 0.25) (2 RCTs)
Kraemer et al. (35) 2022	QoL (FACT-C, EORTC QLQ-C30, TOI, BFI) FC (6MWT, Modified Balk Treadmill & Tread Walk tests)	Home-based or supervised or mixed exercise interventions Comparator: usual care	13 RCTs Search date: 10 March 2021 Meta-analysis: Yes (11 RCTs)	PwCRC regardless of treatment status (*n* = 706)	Home-based exercise intervention: 1. No significant improvement in QoL (SMD = 0.24, 95% CI: –0.22, 0.70, *p* = 0.31) (3 RCTs, *n* = 203) & FC (SMD = 0.21, 95% CI: –0.02, 0.44, *p* = 0.07) (4 RCTs, *n* = 327) 2. Combined data: no significant effect (p = 0.05) (5 RCTs) 3. Intervention adherence ≥80% improves overall QoL & FC (SMD = 0.30, 95% CI: 0.06, 0.54, *p* = 0.01) (3 RCTs, *n* = 272) Supervised or mixed exercise intervention: 1. No significant improvement in QoL (SMD = 0.55, 95% CI: –0.04, 1.14, *p* = 0.07) (3 RCTs, *n* = 162) 2. Significant increase in FC (SMD = 0.45, 95% CI: 0.04, 0.86, *p* = 0.03) (5 RCTs, *n* = 154) 3. Combined data: supervised exercise intervention is effective in improving FC & QoL (*p* = 0.002) (6 RCTs, *n* = 316) 4. Intervention adherence > 80% improves overall QoL & FC (SMD = 0.75, 95% CI: 0.47, 1.03, *p* < 0.00001) (4 RCTs, *n* = 218)
Singh et al. (36) 2020	Safety, feasibility, health outcomes (QoL, aerobic fitness, fatigue, upper-body strength, lower-body strength, anxiety, depression, sleep, body fat percentage, BMI)	Exercise interventions: aerobic, resistance, combined or other (e.g., yoga) Comparator: usual care	19 RCTs Search date: 1 January 2020 Meta-analysis: Yes	PwCRC regardless of treatment status (*n* = 1,293)	Significant effects for exercise interventions: - Improved QoL (SMD = 0.21, 95% CI: 0.05, 0.37, *p* < 0.01) (11 RCTs, *n* = 641) - Reduced fatigue (SMD = –0.23, 95% CI: 0.01, 0.45, *p* = 0.04) (9 RCTs, *n* = 327) - Improved aerobic fitness (SMD = 0.57, 95% CI: 0.16, 0.98, *p* = < 0.01) (17 RCTs, *n* = 892) - Reduced depression rates (SMD = 0.35, 95% CI: 0.02, 0.67, *p* = 0.04) (3 RCTs, *n* = 180) - Improved sleep (SMD = 0.66, 95% CI: 0.27, 1.05, *p* < 0.01) (3 RCTs, *n* = 106) - Reduced body fat (SMD = 0.51, 95% CI: 0.05, 0.97, *p* = 0.03) (7 RCT, *n* = 422) No effect for anxiety, lower body strength & BMI Subgroup analyses suggest larger benefits (*p* < 0.05) for: - QoL and fatigue for supervised interventions; - aerobic fitness, upper-body strength, reduced body fat when interventions were unsupervised; - QoL, aerobic fitness and reduced body fat for ≥12- week interventions; - aerobic fitness when interventions were during chemotherapy; - aerobic fitness and upper-body strength in trials that involved combined CRC patients No difference in adverse event risk (*p* = 0.92)
Brandenbarg et al. (37) 2018	Fatigue	PA intervention	7 studies, 5 RCTs Search date: Dec 2015 Meta-analysis: Yes (5 RCTs)	PwCRC treated with curative intent (*n* = 630)	PA intervention showed no significant effect on fatigue (SMD = 0.21, 95% CI: –0.07 to 0.49, *p* = 0.14) (5 RCTs, *n* = 554), although a trend for reduced fatigue was observed in all intervention groups
Lund et al. (15) 2020	Primary outcome: physical function Secondary outcomes: physical fitness, psychological well-beings, PA, body composition, cancer- or treatment-related symptoms, safety	Exercise interventions: home-based low-intensity walking programmes + supervised programmes with moderate-to high-intensity resistance training + aerobic exercise	8 RCTs Search date: 20 Dec 2019 Meta-analysis: Yes	Patients > = 70years with CRC and receiving chemotherapy treatment (*n* = 552)	Primary outcomes: - Significantly improved self-reported physical function (SMD = 0.26; 95% CI: 0.04–0.48, *p* = 0.02) (5 RCTs, *n* = 330) - No effect on aerobic capacity (SMD, 0.16; 95% CI: –0.21 to 0.52; *p* = 0.39) (5 RCTs, *n* = 125) Secondary outcomes: - Significant improvement in global QoL (SMD = 0.24; 95% CI: 0.02–0.46, *p* = 0.03) (6 RCTs, *n* = 369) - Significant effect for reduced fatigue (SMD = 0.49; 95% CI: –0.79 to –0.19, *p* = 0.00) (5 RCTs, *n* = 182) - No significant effect on depression (SMD, 0.23; 95% CI: – 0.14 to 0.6, *p* > 0.05) (3 RCTs, *n* = 305), PA, body composition, chemotherapy completion - No adverse events reported
McGettigan et al. (18) 2020	Primary outcomes: physical function (Karnofsky Performance Status Scale, ECOG, TUG); disease-related mental health (e.g. HADS, BDI) Secondary outcomes: overall survival, recurrence-free survival, physical fitness, CRF, anthropometry, HRQoL, PA	PA interventions: supervised interventions (3 RCTs), home-based self-directed interventions (5 RCTs), combined supervised and self-directed programmes (7 RCTs) Comparator: usual care or no PA intervention	16 RCTs & cluster-RCTs Search date: June 2019 Meta-analysis: Yes	PwCRC (non-advanced) CRC who are treated surgically or with neoadjuvant or adjuvant therapy (*n* = 992)	Primary outcomes: 1. Physical function: - No evidence at short-term follow-up ( > 12 weeks < 6 months) 2. Disease-related mental health: - No effect for anxiety (SMD = –0.11, 95% CI: –0.40 to 0.18; *p* > 0.05) and depression (SMD = –0.21, 95% CI –0.50 to 0.08, *p* > 0.05) at short- or medium-term follow-up (4 RCTs, *n* = 198) Secondary outcomes: 1. Improved aerobic fitness component of physical fitness at immediate-term follow-up (SMD = 0.82, 95% CI: 0.34 to 1.29, *p* = 0.0007) (7 RCTs, *n* = 295); low-quality evidence 2. Improved CRF at immediate-term follow-up (SMD = 2.16, 95% CI: 0.18 to 4.15; *p* = 0.03) (6 RCTs, *n* = 230); low-quality evidence 3. Improved HRQoL at immediate-term follow-up (SMD = 0.36, 95% CI: 0.10 to 0.62; *p* = 0.007) (6 RCTs, *n* = 230); moderate-quality evidence 4. No effect for hand grip strength, physical flexibility, anthropometric measures (weight, BMI, waist measurement, waist-to-hip ratio, body fat percentage) & PA No RCTs reported overall survival, recurrence-free survival or long-term follow up.
Abdul Razak et al. (70) 2024	Primary outcomes: HRQoL (FACT-C, FACT-F) Secondary outcomes: fatigue, depression, anxiety, sleep quality	Exercise interventions Comparator: not reported	7 RCTs Search date: Not reported Meta-analysis: Yes (6 RCTs)	CRC survivors (*n* = 529)	Primary outcomes: 1. No significant effect of exercise intervention on HRQoL in the intervention group (SMD = 0.25; 95% CI: –0.0, 0.51; *p* = 0.06) (6 RCTs, *n* = 379) Secondary outcomes: 1. No significant improvement in fatigue in intervention group (SMD = 0.11, 95% CI: –0.15, 0.38, *p* = 0.40) (4 RCTs, *n* = 228) 2. No significant improvement in depression in intervention group (SMD = –0.12, 95% CI: –0.36, 0.12; *p* = 0.314) (3 RCTs, *n* = 276) or anxiety (SMD = –0.14; 95% CI: –0.43, *p* = 0.314) (2 RCTs, *n* = 205)
Su et al. (71) 2024	Primary outcomes: physical activities (PA), HRQoL Secondary outcomes: Cancer‑ or treatment‑related symptoms (fatigue, depression, sleep problems)	Internet-based digital health exercise interventions Comparator: usual care without any Internet-related equipment or material	8 RCTs Search date: Dec 2022 Meta-analysis: Yes	CRC survivors (*n* = 1,866)	Primary outcomes: 1. Internet-based intervention led to significant improvements in PA at 6 months (SMD = 0.23; 95% CI: 0.09, 0.38, *p* = 0.001) (2 RCTs, *n* = 762). No significant effect of interventions noted on objective PA at 3 months (SMD = 0.38, 95% CI: – 0.18, 0.93; *p* = 0.19) (2 RCTs, *n* = 133) 2. Significant improvement in HRQoL in the intervention group at 6 months (SMD = 0.11; 95% CI: 0.01, 0.22; *p* = 0.03) (3 RCTs, n = 1387), but no significant difference observed at 3 months (SMD = 0.08; 95% CI: – 0.04, 0.19; *p* = 0.18) (3 RCTs, *n* = 1,153) Secondary outcomes: 1. Significant improvement in fatigue in the intervention group at 6 months (SMD = –0.18, 95% CI: –0.32, –0.04; *p* = 0.01) (2 RCTs, *n* = 747), but no improvement at 3 months (SMD = –0.26; 95% CI: –0.61, 0.08; *p* = 0.14) (2 RCTs, *n* = 747) 2. No significant improvement in intervention group in depression (MD = –0.99, 95% CI: –2.54, 0.55; *p* = 0.21) (2 RCTs, *n* = 747) or anxiety (MD = –0.97; 95% CI: –2.97,1.03; *p* = 0.34) (2 RCTs, *n* = 747)
Nakashima et al. (72) 2023	Primary outcomes: FI, HR-QoL, adverse events Secondary outcomes: bowel dysfunction after surgery	PFMT alone or in combination with patient education, biofeedback, electrostimulation or rectal balloons Comparator: no treatment, UC or usual rehabilitation	7 RCTs Search date: 12 Jan 2023 Meta-analysis: Yes	PwCRC post-surgery (*n* = 252)	Primary outcomes: 1. PFMT showed little to no difference in FI (MD = 0.62; 95% CI: –1.26, 2.5, *p* = 0.52) (2 RCTs, *n* = 148) 2. No trend toward positive impact on HR-QoL (2 RCTs) 3. Little to no difference in adverse events (RR 5.78; 95% CI: 0.28, 177.22) (2 RCTs, *n* = 199) Secondary outcomes: 1. Uncertain effect of PMFT in PwCRC after surgery on bowel dysfunction (SMD = –0.16; 95% CI –0.24, 2.47; *p* = 0.91) (2 RCTs, *n* = 199)
Pun et al. (73) 2024	Primary outcomes: FI measured by anorectal manometry (ARP, MSP, RRP); QoL	Physiotherapy interventions (acupuncture, biofeedback, electrical stimulation, aerobic exercises, resistance exercises, stretching, manual therapy, PFMT, or yoga) Comparator: no treatment, UC, placebo, active control	10 RCTs Search date: Nov 2022 Meta-analysis: Yes	PwCRC post-surgery (*n* = 608)	Primary outcomes: 1. Statistically significant improvements in QoL components including lifestyle (WMD = 0.54; 95% CI 0.03, 1.05; *p* = 0.04), coping behaviour (WMD 1.14; 95% CI 0.24, 2.04; *p* = 0.01), embarrassment (WMD = 0.417; 95% CI 0.14, 0.70; *p* = 0.00) in those receiving PFMT compared with UC (2 RCTs, *n* = 112) 2. Biofeedback alone shown to be more effective than UC in enhancing ARP (WMD 9.55; 95% CI 2.60, 16.51; *p* = 0.01) (3 RCTs, *n* = 226), MSP (WMD 25.29; 95% CI 4.08, 48.50; *p* = 0.02) (3 RCTs, *n* = 226), and RRP (WMD 0.51; 95% CI 0.10, 0.9; *p* = 0.02) (2 RCTs, *n* = 152) 3. PFMT combined with biofeedback shown to be significantly more effective than PFMT alone for ARP (WMD 3.00; 95% CI 0.40, 5.60; *p* = 0.02) (3 RCTs, *n* = 211), MSP (WMD 9.35, 95% CI 0.17, 18.53; *p* = 0.05) (3 RCTs, *n* = 211), and RRP (WMD 1.54; 95% CI 0.60, 2.48; *p* = 0.00) (2 RCTs, *n* = 135)
**Nutritional interventions**					
Liu et al. (38) 2016	Intestinal mucosa integrity measured by lactulose to mannitol ratio (4 RCTs); *Bifidobacterium* to *Escherichia* ratio (3 studies), occludin level (3 RCTs), bacterial translocation rates (3 RCTs), and levels of sIgA (4 RCTs), IL-6 (5 RCTs), and CRP (10 RCTs)	Assortment of pro/synbiotic used pre-operatively or postoperatively Comparator: varies by study and can include intestinal cleaning, unspecific placebo	17 RCTs Search date: April 2015 Meta-analysis: Yes	Patients with elective colorectal surgery (n = 1242)	1. Lactulose/mannitol test: significant reduction in intestinal permeability (SMD = 3.83, *p* = 0.000) (4 RCTs) 2. Occludin: significantly higher occludin and better intestinal mechanical barrier function (SMD = 4.74, *p* = 0.000) (3 RCTs) 3. *Bifidobacterium*/*Escherichia* ratio: significantly higher B/E suggesting higher microbial colonization resistance (SMD = 3.91, *p* = 0.000) (2 RCTs) 4. Bacterial translocation: significantly lower in pro/synbiotics group (*p* = 0.002) (3RCTs) 5. Secretory IgA: Significantly higher in pro/synbiotics group (SMD = 2.91, *p* = 0.004) (3 RCTs) 6. IL-6: No significant differences (SMD = 1.33, *p* = 0.184) (3 RCTs) 7. CRP: significant reduction in CRP in pro/synbiotics group (SMD = 4.21, P = 0.000) (9 RCTs)
Wierzbicka et al. (39) 2021	Changes in gut microbiota/postoperative outcome and selected biochemical and inflammatory parameters (i.e., hsCRP, IL-2, haemoglobin)	Probiotics treatment duration > 6 days Comparator: not specified.	6 RCTs Search date: Jan 2021 Meta-analysis: No	PwCRC with tumour localized in colon (*n* = 457)	1. Probiotic supplementation is associated with increased positive bacteria such as *Bifidobacterium and* reduced pathogenic bacteria such as *Enterococcus* (5 RCTs) 2. Specific probiotic strains e.g., *Bifidobacterium longum* supplements increased serum haemoglobin, erythrocyte, lymphocyte, total protein/albumin concentration, and decreased hsCRP 3. Synbiotic administration increased barrier function, production of INF-gamma and decreased IL-2 secretion 4. Probiotic intervention with *Lactobacillus plantarum*, *Lactobacillus acidophilus*, *Bifidobacterium longum* lowered postop bacterial translocation and increased mean colon mucosal transepithelial resistance; also reduced transmucosal permeation 5. Pre/postsurgical probiotics supplementation improved peristalsis, and reduced postop abdominal symptoms, pyrexia, superficial incisional infections and time to first flatus, and reduced hospital LOS
Dikeocha et al. (40) 2022	Primary outcome: modulation of gut microbiota (6 RCTs); inflammatory biomarkers (3 RCTs) Secondary outcome: postop complications, hospital LOS, QoL, mortality	Mixture of probiotics (12 RCTs), synbiotics (7 RCTs), single probiotics (3 RCTs), kefir as probiotic source (1 RCT) Comparator: Placebo or healthy group control	23 RCTs Search date: Jan 2020 Meta-analysis: No	PwCRC (*n* = 2,457)	Primary outcomes: 1. Modulation of gut microbiota: - Reductions in pathogenic bacteria such as *Fusobacterium* by 6-fold in probiotic group (1.91% of total bacteria vs 10.08% originally; *p* = 0.03) - High dose probiotics was associated with a significantly higher count of *Lactobacilli* cultured in stool samples and lower colonic mucosa adherence levels for *Enterobacteriaceae* 2. Inflammatory biomarkers: - Probiotics and o-3FA significantly decreased the amount of IL-6 (*p* = 0.002); significantly lower serum levels of IL-12 (*p* = 0.005), IL-22 (*p* = 0.018), IL-17A (*p* = 0.00), TNF-a (*p* = 0.002), IL-17C (*p* = 0.018), and IL-10 (*p* = 0.028) in patients as compared with their baseline levels
					Secondary outcomes: 1. Reduction of postoperative complications: - 4 RCTs reported less SSI - 12 RCTs reported no statistical difference in the non-infectious complications 2. Significantly lower rate of bacterial translocation in the probiotic group (*p* = 0.026) 3. 4 RCTs reported no significant effect for hospital LOS: 4. Improvements in QoL Measures (*p* = 0.04) after 12 weeks of treatment
Amitay et al. (41) 2020	Patient well-being, disease burden, postoperative complication rates, prognosis	Prebiotics or synbiotics	16 RCTs Search date: Jan 2020 Meta-analysis: Yes (11 RCTs)	Patients undergoing/completed CRC surgery (n = 1,318)	1. Septicaemia/sepsis: significantly lower incidence (OR = 0.31, 95% CI: 0.18, 0.55, *p* < 0.001) (5 RCTs, *n* = 572) 2. Infection: significantly lower incidence (OR = 0.34, 95% CI: 0.21,0.54, *p* < 0.001) (9 RCTs) 3. Diarrhoea: significantly lower incidence (OR = 0.38, 95% CI: 0.24,0.60, *p* = 0.001) (4 RCTs) 4. LOS: Shorter but not statistically significant (MD = –0.41, 95% CI –0.90, 0.09, *p* = 0.110) (6 RCTs) 5. Return to normal gut function/first defecation: statistically significant shorter duration (MD = –0.66, 95% CI: –0.93, 20.39, *p* < 0.001) (4 RCTs) 6. Antibiotic use: statistically significant shorter duration of antibiotic use (MD = –0.64, 95% CI: –0.83, –0.44, *p* < 0.001) (4 RCTs)
Khan et al. (42) 2023	Postop infectious complications (wound, surgical, bacteraemia, UTI, pneumonia, etc.), SSI, GI anastomotic leak, non-infectious complications, LOS, mortality	IMN administered (pre/peri/postop) including at least 2 of arginine, glutamine, omega3 fatty acids, or nucleotides Comparator: normal diet/non-immune modulating enteral supplements	37 RCTs; 6 RCTs involves CRC Search date: May 2022 Meta-analysis: Yes	Adults undergoing surgery for GI cancer. (*n* = 3,793, *n*(CRC) = 841)	1. Infectious complications: - Overall, in patients with GI cancer, significant reduction in infectious complications was observed after IMN intervention (OR 0.58, 95% CI: 0.47, 0.72, *p* < 0.0001) (33 RCTs) - CRC cancer relation: significantly reduced infectious complications (OR = 0.47, 95% CI 0.27, 0.81, *p* = 0.007) (6 RCTs, *n* = 841) 2. SSI: reduction in SSI (OR = 0.65, 95% CI 0.52, 0.81, *p* = 0.0002) (30 RCTs) 3. Non-infectious complications: no statistically significant effects observed (37 RCTs) 4. GI anastomotic leak rate: significant reduction of leak in peri-op IMN (OR 0.50, 95% CI 0.28, 0.89, *p* = 0.02) but not seen in pre/postop 5. LOS: Significantly reduced after IMN (MD = –1.94, 95% CI: –3.00 to – 0.87, *p* = 0.0004) (29 RCTs) 6. Mortality: no statistical significance
Shen et al. (14) 2022	Overall complications, infectious complications, non-infectious complications, mortality, LOS, enteral nutrition-related complications	Enteral immunonutrition including arginine, glutamine, omega3 fatty acids, nucleotides Comparator: standard diet (isocaloric/isonitrogenous enteral nutrition supplement) or no supplement	35 RCTs (*n*(CRC) = 7 RCTs) Search date: Jan 2022; Meta-analysis: Yes	Patients undergoing surgery for GI cancer (*n* = 3,692; *n*(PwCRC) = 905)	Overall, for patients with GI cancer: - IMN significantly decrease incidence of overall complications (RR = 0.79, 95% CI: 0.70,0.88; *p* < 0.001), significantly lower the incidence of infectious complications (RR = 0.66, 95% CI: 0.55, 0.78; *p* < 0.001) and shorter LOS No effects observed for non-infectious complications or mortality; no difference in IMN-related complications between groups
					For PwCRC: 1. Significantly reduced LOS (RR = –1.74, 95% CI: –3.23, –0.25; *p* = 0.02) (3 RCTs) 2. Significantly reduced incidence of infectious complications (RR = 0.5, 95% CI: 0.36, 0.70; *p* < 0.001) (6 RCTs) 3. Reduced SSI (RR = 0.43, 95% CI: 0.22, 0.81; *p* = 0.009) (6 RCTs) 4. No significant difference in non-infectious complications rates (RR = 1.15, 95% CI: 0.71, 1.87, *p* = 0.58) (3 RCTs)
Bruns et al. (43) 2018	Primary outcome: overall complication rate Secondary outcomes: incision infection rate, anastomotic leakage rate, LOS, QoL, recovery, compliance	Oral nutritional support as macronutrients (proteins, carbs, fats) and micronutrients (immunonutrition, vitamin supplements), or dietary advice administered 48 hours pre-operatively. Comparator: regular diet without nutritional support specifically	5 RCTs/1 controlled trial Search date: 30 Aug 2016 Meta-analysis: Yes	Patients > = 60 years and undergoing CRC surgery (*n* = 583)	1. Overall complication rate: no significant effect (OR = 0.82; 95% CI: 0.52, 1.25, *p* = 0.34) (5 RCTs, *n* = 387) 2. SSI: no effect for pre-op nutritional support (OR 0.57; 95% CI 0.30,1.09, *p* = 0.54) (5 RCTs, *n* = 411) 3. Anastomotic leakage rate: no difference; 0–12% in nutrition groups; 0–10% in control groups (3 RCTs, no meta-analysis) 4. LOS: reduced LOS in nutrition group (MD = 7.6–12.8 vs MD = 6.8–17.8 in control group) (4 RCTs, no meta-analysis) 5. Other outcomes: no significant differences in QoL (2 RCTs), functional walking distance/changes in leak body mass (1 RCT) or weight loss (1 RCT) (*p* > 0.05) 6. Compliance rates ranged from 72–100% in intervention group (4 RCTs)
Xu et al. (44) 2018	Clinical outcome indicators, laboratory index (T cell subsets, cytokines, immunoglobin, biochemical indices)	Immunonutrition including enteral immunonutrition (EIN) and parenteral immunonutrition (PIN) containing arginine, RNA n3FA, glutamine etc. (varies between studies) Comparator: standard enteral nutrition (EN) and parenteral nutrition (PN)	9 RCTs Search date: April 2017 Meta-analysis: Yes	PwCRC who received surgery (n = 1,004)	EIN: 1. Clinical outcome indicators after surgery: - Significantly shorter LOS (MD = 2.35, 95% CI: 1.29, 3.41); *p* < 0.000) (2 RCTs, *n* = 200) - Significantly reduced infectious complications (OR = 0.33, 95% CI: 0.21,0.53, *p* < 0.000) (4 RCTs, *n* = 566) - Significantly reduced SSI (OR = 0.25, 95% CI: 0.11, 0.58, *p* = 0.001) and superficial/deep incisional infections (OR = 0.27, 95% CI: 0.12,0.64, *p* = 0.003) (2 RCTs, *n* = 311) - No significant effects for anastomotic leak, ileus, organ infections, UTIs, respiratory infections, readmission rates PIN: 1. Clinical outcome indicators after surgery - Significantly reduced LOS (MD = 2.66, 95% CI: 0.62, 4.76, *p* = 0.01) (2 RCTs, *n* = 98) 2. Laboratory index - Decreased serum CD8 (MD –4.32, 95% CI: –7.09, –1.55, *p* = 0.002) and decreased IL-6 level (MD = 6.09, 95% CI:–10.11, –2.07, *p* = 0.003) at 1–week postop - Significantly increased CD3 level (MD = 7.50, 95% CI: 3.57, 11.43, *p* = 0.0002), increased CD4/CD8 (MD = 0.50, 95% CI: 0.22, 0.78, *p* = 0.0005), and increased CD4 (MD = 5.47, 95% CI: 2.54, 8.40, *p* = 0.0002) at 1-week postop - Increased CD4 level (MD = 7.59, 95% CI: 3.97, 11.22, *p* < 0.0001) (3 RCTs, *n* = 81)
Jiang et al. (12) 2020	LOS, infectious complications, non-infectious complications, anastomotic leakage	Immunonutrition including glutamine supplements, n-3 PUFA supplements and arginine-based immunonutrition Comparator: other active immunonutrition formulas or standard nutrition	12 RCTs Search date: 15 Oct 2019 Meta-analysis: Yes	PwCRC scheduled for surgery (*n* = 1,032)	1. LOS: - Significantly shortened LOS were observed in glutamine group (OR = –3.91 95% CI: –6.33 to –1.69), arginine-based IMN group (OR = –3.28, 95% CI: –6.31 to –0.45) and n-3 PUFAs group (OR = –3.49, 95% CI: –5.96 to –1.00) (11 RCTs, *n* = 1,006) 2. Infectious complications: - Arginine significantly reduced infectious complications (OR = 0.43 95% CI 0.17 to 0.95) (10 RCTs; *n* = 1,100) 3. Non-infectious complications: - Reduced non-infectious complications rates were observed in glutamine group (OR = 0.07 95% CI 0.00 to 0.78), n-3 PUFAs group (OR = 0.05, 95% CI 0.00 to 0.83) and arginine-based IMN group (OR = 0.08 95% CI 0.00 to 0.99) (6 RCTs, *n* = 890) 4. Anastomotic leakage: no effect observed (8 RCTs; *n* = 1,002) Ranking of different formulas of immunonutrition: - Glutamine worked best for preventing infectious complications and reducing LOS, followed by n-3 PUFAs, arginine-based formulas, standard nutrition in order - Glutamine was least associated with postop non-infectious complications/anastomotic leakage, then arginine-based nutrition, standard formula, and n-3 PUFAs
Yue at al. (45) 2022	Primary outcome: Immune function-related indices (IgA, IgG, IgM, CD3+, CD4+, CD8+, and ratio of CD4+/CD8+) Secondary outcome: nutritional status-related indices (TP, ALB, and prealbumin)	Addition of omega-3 PUFAs to the control group treatment Comparator: conventional nutrition or blank treatment (fluid supportive therapy)	20 RCTs Search date: 10 April 2022 Meta-analysis: Yes	PwCRC who underwent radical surgery (*n* = 1,613)	Primary outcome: 1. Humoral immune function indices: - Significantly increased IgA (SMD = 0.54, 95% CI: 0.10, 0.99, *p* = 0.000) (7 RCTs, *n* = 478) - Significantly increased IgM (SMD = 0.52, 95% CI: 0.05, 0.99, *p* = 0.000) (6 RCTs, *n* = 437) - Increased IgG (SMD = 0.65, 95% CI 0.47–0.84, *p* = 0.495) (7 RCTs, *n* = 477) 2. T cell immune function indices: - Significantly increased CD4+ (SMD = 0.76, 95% CI: 0.53, 0.98, *p* = 0.023) (10 RCTs, *n* = 746) - Significantly higher ratio of CD4+/CD8+ (SMD = 0.66, 95% CI: 0.39, 0.92, *p* = 0.011) (9 RCTs, *n* = 626) - Markedly reduced CD8+ (SMD = –0.28, 95% CI: –0.66, 0.09, *p* = 0.000) (9 RCTs, *n* = 678) - Increased CD3+ (SMD = 0.73, 95% CI 0.54– 0.92, *p* = 0.833) (7 RCTs, *n* = 449) Secondary outcome: Nutritional status indicators: - Significantly increased TP (SMD = 0.53, 95% CI: 0.17, 0.88, *p* = 0.000) (9 RCTs, *n* = 762) - Significantly increased ALB (SMD = 0.43, 95% CI: 0.15, 0.70, *p* = 0.000) (15 RCTs, *n* = 1,278) - Significantly increased prealbumin (SMD = 0.46, 95% CI: 0.01, 0.90, *p* = 0.000) (11 RCTs, *n* = 933)
Li et al. (46) 2023	Postoperative complications, immune regulation, inflammatory factors, weight/BMI	PUFA supplementation via intravenous/oral administration +/- co-intervention with immune nutrients like arginine/nucleotides Comparator: placebo/reagents without PUFAs	12 RCTs Search date: January 2022 Meta-analysis: Yes	PwCRC who underwent surgery/chemotherapy (*n* = 702)	1. Postoperative complications - Overall, no effect on total infectious complications (RR = 0.72, 95% CI: 0.39, 1.32, *p* = 0.29) (6 RCTs, *n* = 491) or LOS (WMD = –1.19, 95% CI: −2.62, 0.24, *p* = 0.10) - Subgroup: Preop supplementation reduced infectious complications (RR = 0.37, 95% CI: 0.16, 0.85, *p* = 0.02) (2 RCTs *n* = 167) and shortened LOS (WMD = –2.27, 95% CI: –3.58, –0.97, *p* < 0.001) - No effect for perioperative intervention for reduced infectious complications (RR: 0.98, 95% CI: 0.42, 2.28, *p* = 0.96) (4 RCTs; *n* = 317) and shortened LOS (WMD = –0.08, 95% CI: –1.97, 1.80, *p* = 0.93) 2. Immune regulation - Improved CD4/8 ratio but was not statistically significant (WMD = 0.32, 95% CI: –0.03, 0.68, *p* = 0.07) - No effect on CD4 and CD8 level 3. Inflammatory factors/weight/BMI - Significantly increased albumin levels (WMD = 0.48, 95% CI: 0.04, 0.91, *p* = 0.03) (3 RCTs, *n* = 110) - Reduced CRP levels (WMD = –6.12, 95% CI: –11.31, –0.92, *p* = 0.02) (2 RCTs; *n* = 113) - Reduced TNF-alpha (SMD = –0.56, 95% CI: –0.97, –0.15, *p* = 0.007) - Reduced IL-6 in chemotherapy group (SMD = –0.53, 95% CI: –1.15, 0.08, *p* = 0.09) and the surgery group (SMD = –0.64, 95% CI: –1.05, –0.23, *p* = 0.002) - Significantly reduced IL-6 levels (SMD = –0.54, 95% CI: –0.91, –0.17, *p* = 0.004) - No effect on IL-1beta, weight, and BMI
Ye et al. (47) 2023	Inflammatory indicators (CRP, TNF-α, IL-6); nutritional indicators (Alb, weight, BMI); clinical outcomes (LOS, UTI, pneumonia)	Glutamine, arginine, omega3, probiotics, vitamin D, omega3/arginine combined, omega3/vitamin D combined, omega3/probiotics combined Comparator: Placebo	34 RCTs Search date: Dec 2022 Meta-analysis: Yes (Network meta-analysis)	PwCRC who underwent chemotherapy or chemoradiotherapy (*n* = 2,841)	Inflammatory indicators: 1. TNF-α: significantly reduced levels found after glutamine (MD = –25.2; 95% CrI (–32.62, –17.95)) and probiotics (MD = –12.55; 95% CrI: –15.19, –9.92) (8 RCTs); glutamine most effective (SUCRA = 99.9%) 2. IL-6: Significantly reduced IL-6 found after omega3/arginine (MD = –61.41; 95% CrI: – 97.85, –24.85) and probiotics (MD = – 21.12; 95% CrI: –40.38, –2.89) (14 RCTs); Omega3/arginine best to reduce IL-6 (SUCRA = 98.6%) 3. CRP: No significant effect (9 RCTs); Vitamin D may be best to reduce CRP (SUCRA = 87.2%) Nutritional indicators: 1. Most supplements reduced Alb, weight, BMI levels but were not statistically significant (6 RCTs); Omega3/vitamin D may be best to maintain Alb (SUCRA = 76. 9%) and BMI (SUCRA = 63.6%) Clinical outcomes: 1. LOS: glutamine (MD = –3.71; 95% CrI = –5.89, –1.72) and omega-3 (MD = –3.41, 95% CrI (–6.03, –0.81)) significantly reduced the LOS (13 RCTs) - Glutamine has highest evidence (SUCRA = 78.7%) 2. UTIs: no effect (9 RCTs) - Probiotics has highest evidence (SUCRA = 83.5%)
					3. Wound infections: glutamine and probiotics both reduced risk of infections by 88% (RR = 0.12; 95% CrI (0, 0.85)) and 39% (RR = 0.61; 95% CrI (0.41, 0.86)), respectively - Similar advantage found in glutamine compared with omega3 (RR 0.11; 95% CrI (0, 0.94) - Glutamine best for reduction of infections (SUCRA = 94.5%) 4. Anastomotic leaks: no significant findings (12 RCTs) - Glutamine may be best for reduction (SUCRA = 84.1%) 5. Pneumonia: probiotics reduced risk by 62% (RR 0.38; 95% CrI 0.15, 0.81) (SUCRA = 82.0%) (10 RCTs)
Veziant et al. (48) 2023	Primary outcomes: Overall infectious complications and SSIs including both deep abdominal infections and wound infections Secondary outcomes: anastomotic leaks, wound infections, UTI, pulmonary infections	Synbiotics or probiotics administered preoperatively and/or postoperatively Comparator: placebo or standard care	21 RCTs Search date: 14 Feb 2022 Meta-analysis: Yes	PwCRC who underwent surgery (*n* = 1,961)	Primary outcomes: 1. Significantly fewer infectious complications (RR = 0.59, 95% CI: 0.47, 0.75, *p* < 0.01) (12 RCTs, *n* = 1,299) 2. Significantly fewer SSI (RR = 0.70, 95% CI: 0.52, 0.95, *p* = 0.02) (11 RCTs, *n* = 1,297) Secondary outcomes: 1. Significantly fewer pulmonary infections (RR = 0.35, 95% CI 0.20, 0.63, *p* < 0.01) (10 RCTs) 2. Significantly fewer UTI (RR = 0.41, 95% CI: 0.19, 0.87, *p* = 0.02) (6 RCTs) 3. No significant effect for anastomotic leaks (RR = 0.83, 95% CI: 0.47, 1.48, *p* = 0.53) and wound infections (11 trials, RR 0.74 (0.53–1.03), *p* = 0.08) (11 RCTs) Probiotics vs synbiotics: - Probiotics have fewer overall infectious complications (RR = 0.55, 95% CI: 0.42,0.73) (9 RCTs) and fewer SSIs (RR = 0.63, 95% CI: 0.44, 0.91) (8 RCTs) - No significant effect for infectious complications (RR = 0.69, 95% CI: 0.42, 1.13) and SSI (RR = 0.87, 95% CI: 0.47, 1.60) (3 RCTs) No significant difference in multi-strain and non-multistrain pro/synbiotics subgroups No significant differences in subgroups based on administration timing (pre vs peri vs postop)
Yang et al. (49) 2021	Humoral immune function, T Cell immune function, postoperative complications (SSI, anastomotic leakage), LOS	Glutamine Comparator: routine nutrition or blank therapy	31 RCTs Search date: 30 July 2021 Meta-analysis: Yes	PwCRC who underwent radical surgery (*n* = 2,201)	1. Humoral immune function: Glutamine significantly improved the humoral immune function indicators: - IgA (SMD = 1.15, 95% CI: 0.72, 1.58, *p* = 0.000) (14 RCTs) - IgM (SMD = 0.68, 95% CI: 0.48, 0.89, *p* = 0.000) (17 RCTs) - IgG (SMD = 1.10, 95% CI: 0.70, 1.50, *p* = 0.000) (17 RCTs) 2. T cell immune function indicators: - Significantly increased CD4+ (SMD = 0.76, 95% CI: 0.53, 0.99, *p* = 0.000) (15 RCTs) - Significantly increased ratio of CD4+/CD8+ (SMD = 0.92, 95% CI: 0.57, 1.28, *p* = 0.000) (13 RCTs) - Significantly decreased CD8+ (SMD = –0.50, 95% CI: –0.91, –0.10, *p* = 0.015) (15 RCTs)
					3. Postop complications: - Significantly reduced SSI (RR = 0.48, 95% CI: 0.30, 0.75, *p* = 0.001) (12 RCTs) - Significantly reduced anastomotic leak (RR = 0.23, 95% CI: 0.09, 0.61, *p* = 0.003) (7 RCTs) - Significantly reduced LOS (SMD = – 1.13, 95% CI: –1.68, –0.58, *p* = 0.000) (8 RCTs)
Jolfaie et al. (50) 2015	Postoperative symptoms, immunity (T cell dysfunction)	Glutamine in oral or supplemental or injection or dietary form Comparator: routine care	9 RCTs Search date: July 2015 Meta-analysis: No	PwCRC undergoing chemotherapy or radiochemotherapy or surgery (*n* = 418)	PwCRC undergoing chemotherapy: - Gln increased ratio of villus height to crypt depth in duodenum/stomach and decreased incidence of gastric/duodenal mucositis (1 RCT) - Oral Gln reduced neuropathy (1 RCT) and stomatitis/neutropenia (1 RCT) - Gln supplement prevented intestinal mucositis, reduced diarrhoea, improved intestinal absorption and intestinal permeability induced by 5-FU (1 RCT) PwCRC undergoing chemoradiotherapy: No significant difference in frequency/severity of diarrhoea/inflammatory levels (1 RCT) PwCRC undergoing surgery: - Gln lowered rate of SSI, intraabdominal abscess formation, wound dehiscence, and LOS (1 RCT) - Gln increased T cell synthesis; no effect on IL2/6/TNF-alpha levels (1 RCT) - Gln improved immune response, lymphocyte recovery, nitrogen balance, and reduced LOS (1 RCT) - No effect for gastrointestinal complications post-surgery (1 RCT)
Liu et al. (51) 2023	TNF-α, CRP, IL-6, IL-1β, albumin, BMI, weight, infectious and non-infectious complication rates, LOS, mortality, QoL	O3 FA via all routes (oral, PN, TPN, EN), alone or in combination with other parenteral/enteral feeding regimes	19 RCTs Search date: March 2023 Meta-analysis: Yes	PwCRC undergoing surgery and/or receiving chemo (*n* = 1,556)	Inflammatory factors: 1. Significantly reduced TNF-α (MD = –0.79, 95% CI: –1.51, –0.07, *p* = 0.03) (8 RCTs, *n* = 432); 2. No significant effect for CRP (MD = –2.41, 95% CI: –5.45, 0.63, *p* = 0.12) (5 RCTs, *n* = 294) 3. Significantly reduced IL-6 (MD = –4.70, 95% CI: –6.59, –2.80, *p* < 0.00001) (7 RCTs) 4. No statistical effect for IL-1β (MD = 0.31, 95% CI: –0.41 to 1.02, *p* = 0.40) (3 RCTs) Nutritional status: 1. No effect for ALB (MD = 0.31, 95% CI: –0.10 to 0.71, *p* = 0.14) (4 RCTs, n = 154) 2. No effect for BMI (MD = –0.19, 95% CI: –1.46 to 1.09, *p* = 0.77) (4 RCTs) 3. No effect for weight (MD = 0.54, 95% CI: –3.46 to 4.54, *p* = 0.79) (5 RCTs) 4. No effect for rates of non/infectious complications (RR = 0.96, 95% CI: 0.65, 1.42, *p* = 0.83) (9 RCTs, n = 715) But lower rates of infectious and non-infectious complications with PN route (RR = 3.73, 95% CI: 1.52 to 9.17, *p* = 0.004) 5. Significantly reduced LOS (MD = 9.36, 95% CI: 2.16, 16.57, *p* = 0.01) (5RCTs, n = 647) 6. No effect for mortality (RR = 0.86, 95% CI: 0.28, 2.70, *p* = 0.80) (4 RCTs) 7. No effect for QoL (RR = 9.12, 95% CI: −11.20 to 29.45, *p* = 0.38) (3 RCTs)
Chen et al. (52) 2022	Immunity measured by serum IgG, IgA, IgM, CD4+ T-cells, CD8+ T-cells and CD4+-to-CD8+ ratio	Probiotics intervention used perioperative phase Comparator: routine care	6 RCTs Search date: 2 June 2021 Meta-analysis: Yes	Perioperative CRC patients (not treated with chemo or radiation therapy) (*n* = 492)	1. Humoral immunity: - Significantly increased serum IgG (MD = 1.33; 95% CI: 0.88, 1.77, *p* < 0.000) (4 RCTs, *n* = 310) - Significantly increased IgA (MD = 0.20; 95% CI: 0.10, 0.30; *p* = 0.0002) (4 RCTs, *n* = 310) - Significantly increased IgM (MD = 0.18, 95% CI: 0.13, 0.24; *p* < 0.000) (4 RCTs, *n* = 310) 2. Cellular immunity: - Significantly increased CD4+ (MD = 2.79; 95% CI: 2.34, 3.24, *p* < 0.000) (4 RCTs, *n* = 352) - No effect on CD8+ (MD = 0.21, 95% CI: –0.18, 0.61, *p* = 0.29) (4 RCTs, *n* = 352) - Significantly increased CD4+/CD8+ (MD: 0.09; 95% CI: 0.04, 0.13; *p* = 0.0003) (4 RCTs, *n* = 352)
An et al. (53) 2022	Primary outcomes: perioperative mortality, postoperative ( < 30 postoperative days), infectious complications, probiotics-related adverse events Secondary outcomes: overall postoperative complications, LOS, postoperative QoL	Perioperative probiotics/prebiotics Comparators: placebo or standard care	20 RCTs Search date: 12 August 2022 Meta-analysis: Yes	Patients undergoing open, laparoscopic, or robotic CRC surgery for curative intent (*n* = 1,763)	Primary outcomes: 1. No effect in reducing perioperative mortality (RR = 0.17, 95% CI: 0.02, 1.38, moderate CoE) (8 RCTs, *n* = 753) 2. Significantly lower postoperative infectious complications (RR = 0.45, 95% CI: 0.27 to 0.76, low CoE) (7 RCTs, *n* = 651) 3. Adverse events: little to no difference (RR = 0.73, 95% CI: 0.45, 1.19; I2 = 0%; Low CoE) (7 RCTs) Secondary outcomes: 1. Reduced overall postoperative complications (RR = 0.47, 95% CI: 0.30, 0.74, low CoE) (6 RCTs, *n* = 394) 2. No effect for hospital LOS (MD = –1.06, 95% CI: –1.64, – 0.47; low CoE) (8 RCTs, *n* = 411) 3. Postoperative QoL (MD = 5.64, 95% CI: 0.98 to 10.3; low CoE)
Probst et al. (13) 2017	Mortality, overall complications, infectious complications, LOS	IMN including Gln, arginine, O-3FA, RNA, nucleotides administered before and/or after major abdominal surgery	83 RCTs, (*n*(CRC) = 8 RCTs) Search date: July 2015 Meta-analysis: Yes	Patients who underwent major abdominal surgery *n*(total) = 7,116 *n*(CRC) = 462	Reduced infectious complications (OR = 0.58, CI 0.51, 0.66; *p* < 0.001) and shortened hospital stay (MD = –1.79, 95% CI: –2.3, –1.19, *p* < 0.001), IMN reduced overall complications (OR = 0.79, 95% CI: 0.66, 0.94; *p* = 0.01) CRC dubgroup: no significant effects for infectious complications, LOS, mortality, or overall complications
Xiong et al. (74) 2023	Primary outcomes: plasma proteins including albumin (ALB), prealbumin (PA), nitrogen balance (NB), total protein (TP) Secondary outcomes: inflammatory markers TNF-α, CRP, and infectious complications (ICs)	Glutamine via enteral or parenteral nutrition Comparator: conventional nutrition or blank treatment	26 RCTs Search date: March 2023 Meta-analysis: Yes	PwCRC after curative surgery (*n* = 1,678)	Primary outcomes: 1. Markedly higher ALB contents in glutamine cohort (SMD = 0.79, 95% CI: 0.55, 1.03; *p* = 0.000) 2. Significantly increased prealbumin level in glutamine cohort (SMD = 0.94, 95% CI: 0.69, 1.20; *p* = 0.000) 3. Significantly increased NB level (SMD = 1.11, 95% CI: 0.46, 1.75; *p* = 0.001) 4. No effect on total protein level (SMD = − 0.02, 95% CI: − 0.60, 0.57; *p* = 0.959) Secondary outcomes: 1. Substantial reduction in the content of TNF-α in the glutamine group (SMD = − 1.86, 95% CI: −2.21, −1.59; *p* = 0.000) 2. Significantly decreased CRP (SMD = − 1.94, 95% CI: − 2.41, − 1.48; *p* = 0.000) 3. Significantly decreased incidence of infectious complications in glutamine cohort (RR = 0.31, 95% CI: 0.21, 0.46; *p* = 0.000) (11 RCTs, *n* = 768)
Qin et al. (75) 2024	Primary outcomes: grip strength Secondary outcomes: weight, BMI, prevalence of skeletal sarcopenia	Oral nutritional supplements (ONS) consisting of a certain proportion of carbohydrates, proteins, fats, minerals, and trace elements in addition to the daily diet Comparator: placebo or a regular diet	11 RCTs Search date: Sept 2023 Meta-analysis: Yes	PwCRC undergoing surgery (*n* = 1,070)	Primary outcomes: 1. 4 RCTs reported statistically inconsistent positive effect of ONS on grip strength (very low-quality evidence) Secondary outcomes: 1. 10 RCTs reported positive effect of ONS on long-term postoperative (12–15 weeks) bodyweight or BMI change (very low-quality evidence) - 4 RCTs showed significantly improved bodyweight and BMI. 1 RCT showed both groups lost bodyweight and a decreased BMI, but ONS group lost less weight than control after 15 weeks of intervention. Other 5 RCTs reported a non-significant positive effect of ONS on weight and BMI 2. Sarcopenia - Significantly lower prevalence of postoperative sarcopenia in the ONS group after 3–6 months of intervention (OR = 0.48, 95% CI: 0.29, 0.81; *p* = 0.006) (2 RCTs, *n* = 265) 3. QoL - 6 RCTs reported no significant effect of ONS on QoL
Persson et al.(76) 2024	Primary outcomes: diarrhoea (loose or liquid stools more than 3 times a day) Secondary outcomes: infectious complications; abdominal distention; length of antibiotic therapy; duration of postoperative pyrexia; LOS; time to first defecation and initiation of a solid diet	Perioperative or postoperative microbiota modulation with probiotics Comparator: Placebo	10 RCTs Search date: 18 Aug 2023 Meta-analysis: Yes	PwCRC undergoing surgery (*n* = 1,276)	Primary outcomes: 1. Significant decrease in incidence of diarrhoea in the probiotic group (OR = 0.42; 95% CI: 0.31, 0.55; *p* < 0.001) (6 RCTs, *n* = 952) Secondary outcomes: 1. Infectious complications: - Significant reduction in incidence of SSI (OR 0.44; 95% CI: 0.22, 0.89; *p* = 0.023) (3 RCTs, *n* = 302) - Significant reduction in incidence of urinary infection (OR 0.43; 95% CI: 0.20, 0.91; *p* = 0.028) (4 RCTs, *n* = 488) - Significant reduction in incidence of pulmonary infection (OR 0.30; 95% CI: 0.15, 0.60; *p* < 0.001) (5 RCTs, *n* = 548) 2. Significant reductions in abdominal distention (OR 0.43; 95% CI: 0.25, 0.76; *p* = 0.004) (3 RCTs, *n* = 274); length of antibiotic therapy (MD = − 1.66 days; 95% CI: −2.13, −1.19 days; *p* < 0.001) (3 RCTs, *n* = 364), and duration of postop pyrexia (MD −0.80 days; 95% CI: −1.38, −0.22 days; *p* = 0.007) (3 RCTs, *n* = 364) in the probiotic group 3. No significant difference for LOH stay, time of defecation and time to first solid diet
Zhang et al. (77) 2024	Surgery-related indicators (operation time, blood transfusion, function recovery time, LOS; postoperative complications, nutrition-related indicators (ALB, pre-albumin, transferrin, CRP), immune function, and tumour infiltrative lymphocytes related outcomes (proportion of infiltrative CD4+, CD8+, CD16+, and CD56+ cell subsets in tumour tissue)	Preoperative immunonutrition delivered through enteral channel Control: standard diet or placebo	16 RCTs Search date: May 2023 Meta-analysis: Yes	PwCRC undergoing surgery (*n* = 1,416)	1. Surgery-related indicators: - Patients in the control group had a significantly shorter duration of operation (MD = 15.38, 95% CI: 7.44, 23.31; *p* < 0.01) (3RCTs, *n* = 163) - No difference in blood transfusion rates (RR = 0.81, 95% CI: 0.39, 1.68; *p* = 0.57); (2 RCTs, *n* = 96) - Significantly shorter function recovery time in the intervention group (MD = −21.43, 95% CI: −23.30, −19.55; *p* < 0.01) (2RCTs, *n* = 120) - Decreased LOS in the intervention group but not statistically significant (MD = −0.92, 95% CI: −1.93, 0.08; *p* = 0.76) (10 RCTs, *n* = 1,002) 2. Postoperative complications: - Risk of infection was significantly reduced in the intervention group (RR = 0.53, 95% CI: 0.41, 0.69; *p* < 0.001) (11 RCTs, *n* = 1,081); Subgroup: pre-operation intervention group (RR = 0.56, 95% CI: 0.36, 0.88; *p* = 0.01); pre-operation plus post-operation intervention group (RR = 0.52, 95% CI: 0.38, 0.71; *p* < 0.01) - Significantly reduced SSI in the intervention group (RR = 0.44, 95% CI: 0.27, 0.70; *p* < 0.01) (8 RCTs, *n* = 874) - No difference in readmission rates (RR = – 0.60, 95% CI: 0.27, 1.32; *p* = 0.20) (6 RCTs, *n* = 691) - No difference in anastomotic leak risks (RR = 0.64; 95% CI: 0.36, 1.14; *p* = 0.13) (6 RCTs, *n* = 677) 3. Nutrition-related indicators: - No significant difference in the level of pre-albumin (SMD = 0.90, 95% CI: −0.14, 1.94; *p* = 0.09) (4 RCTs, *n* = 404), ALB (SMD = −0.01, 95% CI: −0.17, 0.15; *p* = 0.88) (6 RCTs, *n* = 606) and transferrin (SMD = 0.49, 95% CI: −0.08, 1.05; p = 0.09) - Significantly lower postoperative CRP in the intervention group (MD = −3.81, 95% CI: −5.48, −2.14; *p* < 0.01) (2 RCTs, *n* = 324) 4. Humoral/cellular immune-related outcomes: - Significantly higher IgA (MD = 0.58, 95% CI: 0.31, 0.85; *p* < 0.01) (4 RCTs, *n* = 240), IgG (MD = 1.67, 95% CI: 1.15, 2.19; *p* < 0.01) (4 RCTs, *n* = 240), and IgM (MD = 0.40, 95% CI: 0.20, 0.61; *p* < 0.01) (3 RCTs, *n* = 200) in the intervention group - No significant difference in CD4 + level (SMD = 2.39; 95% CI: −1.39, 6.18; *p* = 0.22) (2 RCTs, *n* = 160) - No significant difference in CD4+/CD8 + ratio (MD = 0.45; 95% CI: −0.18, 1.09; *p* = 0.16) (2 RCTs, *n* = 160) 5. Tumour infiltrative lymphocytes-related outcomes after intervention: - Administration of immunonutrition did not significantly alter the proportion of CD4 + (Fig. 6A, MD = 0.00, 95% CI: −0.02, 0.02; *p* = 0.94, I2 = 0%) and CD8 + (MD = −0.21, 95% CI: −0.70, 0.29; *p* = 0.41, I2 = 100%) cells in tumour tissues (2RCTs, *n* = 54) - Significantly increased CD16+ (MD = 0.04, 95% CI: 0.02, 0.06; *p* < 0.001) and CD56+ (MD = 0.05, 95% CI: 0.03, 0.06; *p* < 0.001) cell subsets in the intervention group (2 RCTs, *n* = 54)
**Acupuncture** i**ntervention for CRC**					
Liu et al. (54) 2018	Gastrointestinal function recovery	Acupuncture and related therapies (EA, MA, acupressure, moxibustion, point application and laser acupuncture, or any combination) Comparator: sham/placebo acupuncture or no additional intervention	22 RCTs Search date: Oct 2017 Meta-analysis: Yes (21 RCTs)	PwCRC who received surgery (*n* = 1,628)	1. Acupuncture vs sham acupuncture: - Significantly reduced time to first bowel sounds (WMD = –11.41, 95% CI: –20.96, –1.85, *p* = 0.019) (2 RCTs), first flatus (WMD = –15.79, 95% CI: –26.10, –5.49, *p* = 0.002) (4 RCTs), first defecation (WMD = –22.42, 95% CI: –39.14, –5.70, *p* = 0.007) (4 RCTs) 2. Acupuncture vs postoperative care: - Significantly reduced time to first bowel sounds (WMD = –7.76, 95% CI: –9.48, –5.03, *p* = 0.000) (13 RCTs), first flatus (WMD = –14.74, 95% CI: –19.68, –9.79, *p* = 0.000) (17 RCTs), first defecation (WMD = –12.30, 95% CI: –16.71, –7.89, *p* = 0.000) (13 RCTs)
Kim et al. (55) 2016	Primary outcomes: early postoperative symptoms Secondary outcomes: gastrointestinal functional recovery, safety	Acupuncture stimulation style: MA, MA plus EA; MA plus heat application (warming the needle using the moxibustion technique) Comparator: usual care, sham acupuncture or fast-track recovery programme without acupuncture	7 RCTs Search date: June 2015 Meta-analysis: Yes	Participants: patients who underwent CRC resection (*n* = 540)	Primary outcomes: 1. Postoperative pain: no overall benefit at postoperative day 1 (RR = 0.89, 95% CI: 0.47, 1.67) or postoperative day 5 (RR = 1.00, 95% CI: 0.07, 14.55) (4 RCTs) 2. PONV: no significant effect (4RCTs) 3. Sleep: no significant effect (MD = –0.07, 95% CI: –0.90 to 0.76) (1 RCT) 4. Abdominal distension: no significant effect (MD 0.22, 95% CI: –0.34 to 0.78) (1 RCT) Secondary outcomes: - Significantly reduced time to first flatus (hours) (MD = –7.48 h, 95% CI: –14.58 to –0.39) (3 RCTs, *n* = 207) - Significantly reduced time to first defecation (hours) (MD = 18.04, 95% CI: –31.90 to –4.19) (2 RCTs, *n* = 149) - No reported side effects/safety events
Liu et al. (56) 2017	Primary outcomes: time to first flatus and time to defecation Secondary outcomes: time to first bowel sounds, opioids consumption, VAS, LOS	MA, EA and acupressure Comparator: no acupuncture, sham acupuncture, and other active control therapies	10 RCTs Search date: Jan 2017 Meta-analysis: Yes	Adults with cancer (gastric and CRC) who underwent surgery (*n* = 776)	Primary outcomes: 1. Significantly reduced time to first flatus (SMD = –0.98, 95% CI: –1.84, –0.12, *p* = 0.03) (6 RCTs, *n* = 433) 2. Significantly reduced time to first defecation (SMD = –1.23, 95% CI: –2.26, –0.20, *p* = 0.02) (6 RCTs, *n* = 434) Secondary outcomes: 1. Significantly less opioid consumption (SMD = –0.38, 95% CI: –0.59, –0.17, *p* = 0.0005) (4 RCTs, *n* = 409) 2. Shorter time to first bowel sounds but not statistically significant (SMD = –2.35, 95% CI: –4.74 to 0.03, *p* = 0.05) (3 RCTs, *n* = 193) 3. No effect for LOS (SMD = –0.18, 95% CI: –0.46, 0.10; *p* = 0.20) (3 RCTs, *n* = 282) 4. No side effects reported (4 RCTs)
Xu et al. (57) 2021	Primary outcomes: QoL (EORTC, QLQ-CR29) Secondary outcomes: LARS score, adverse events	EA, floating needle, fine needle, etc. or moxibustion at acupoints or trigger points Comparator: no acupuncture, placebo, or other active therapies	6 RCTs Search date: July 2021 Meta-analysis: Yes (2 RCTs, *n* = 200)	PwCRC with defecation dysfunction after sphincter preserving surgery (*n* = 439)	Statistically significant pooled benefits of acupuncture plus biofeedback and electropuncture may improve the EORTC QLQ-CR29 within the following domains: - defecation (MD = –0.39 points; 95% CI: –0.46, –0.32, *p* < 0.00001) (2 RCTs, *n* = 200) - sexual function (MD = –0.71 points; 95% CI: –0.89 to –0.54, *p* < 0.00001) (2 RCTs, *n* = 200) - urination (MD = –0.49 points; 95% CI: –0.77 to –0.20, *p* = 0.0007) (2 RCTs, *n* = 200) - self-feelings (MD = –0.59 points; 95% CI: –0.85, –0.33, *p* < 0.00001) (2 RCTs, *n* = 200) - abdominal pain (MD = 0.93 points; 95% CI: 0.48, 1.38, *p* = 0.002) (2 RCTs, *n* = 200) - stool (MD = 1.04 points; 95% CI: 0.36, 1.73, *p* = 0.003) (2 RCTs, *n* = 200)
Qi et al. (58) 2023	Early postoperative symptoms, PONV, postoperative pain scores	MA, EA or warm-needling Comparator: sham acupuncture or routine nursing	22 RCTs Search date: 28 Feb 2023 Meta-analysis: Yes	PwCRC undergoing abdominal surgery (*n* = 1,878)	1. Significantly improved time to first flatus (MD = –0.77, 95% CI: –1.22 to –0.33, *p* = 0.0007) (11 RCTs, *n* = 876) 2. Significantly improved time to first bowel movement (MD = –1.41, 95% CI: –2.20 to –0.63, *p* = 0.0004 (9 RCTs, n = 671). 3. Significantly improved time to first defecation (MD = –1.03, 95% CI: –1.88 to –0.18, *p* < 0.00001) (9 RCTs, *n* = 556) 4. Significantly less PONV (RR = 0.72, 95% CI: 0.59, 0.89, *p* = 0.002) (5 RCTs, *n* = 1,488) 5. No effect for postoperative pain (MD = –0.21, 95% CI: –0.59 to 0.17, *p* = 0.27) (5 RCTs, *n* = 1,188)
Zhao et al. (59) 2023	Primary outcomes: time to first flatus, time to first defecation Secondary outcomes: time to first bowel motion, LOS	MA and EA Comparator: blank/sham stimulation (B/S) group did not receive treatment or stimulated non-meridian points	13 RCTs Search date: Feb 2023 Meta-analysis: Yes	PwCRC during perioperative period (*n* = 795)	Primary outcomes: 1. Reduced time to the first flatus (SMD = –0.57; 95% CI: –0.73, –0.41, *p* < 0.00001) (10 RCTs) 2. Reduced time to the first defecation (SMD = –4.92 h, 95% CI: –8.10 to –1.74, *p* = 0.002) (7 RCTs) Secondary outcomes: 1. Reduced time to the first bowel motion (MD = –6.62 h, 95% CI: –8.73, –4.50 h, *p* < 0.00001) (7 RCTs) 2. Reduced LOS (SMD = –0.40, 95% CI: –0.60, –0.21, *p* < 0.0001) (6 RCTs) Subgroup analysis (4 RCTs): - In comparison with ERAS group, acupuncture/ERAS reduced time to first flatus (MD = –6.41, 95% CI: –9.34, –3.49, *p* < 0.0001) and time to first defecation (MD = –6.02, 95% CI: –9.28, –2.77, *p* = 0.0003)
**Psychosocial interventions**					
Son et al. (19) 2019	QoL	Psychological support interventions (CBT, psychotherapy, counselling, supportive therapy, motivational interviewing) Social support intervention (social skills training)	8 RCTs Search date: Oct 2016 Meta analysis: Yes	PwCRC (*n* = 2,117)	Statistically significant increase in QoL (Hedges’ G = 0.145, 95% CI: 0.035, 0.254, *p* = 0.009) (8 RCTs, *n* = 2,117) Subgroups: - Face to face: statistically significant improvement (Hedges’ G = 0.160, 95% CI: 0.018, 0.303, *p* = 0.028) (5 RCTs) - non-face to face: no effect (Hedges’ G = 0.116, 95% CI: –0.048, 0.293, *p* = 0.158) (3 RCTs)
Wan et al. (60) 2022	Self-efficacy (NIH PROMIS, CBI-B, GSE, SSES, modified Korean self-efficacy scale), anxiety (HADS or STAI-S), depression, QoL	Web-based psychosocial interventions (psychoeducation, ACT, CBT, peer support, counselling, stress management) Comparator: no intervention or website with non-equivalent intervention contents	19 studies: 12 RCTs and 7 quasi-experimental Search date: Dec 2021 Meta-analysis: Yes (10 RCTs)	PwCRC with smartphone, tablet to internet access (*n* = 1,386)	1. Minimal effect on groups’ self-efficacy favours the control (SMD = 0.93, 95% CI: 0.52, 1.35, *p* < 0.000) (7 RCTs, *n* = 1,002) 2. Minimal effect for QoL (MD = 2.83, 95% CI: –0.31, 5.98, *p* = 0.08) (4 RCTs, *n* = 211) 3. Significantly reduced anxiety - measured by HADS (MD = –2.23, 95% CI: –3.31, –1.14, *p* < 0.01) (3 RCTs, *n* = 177) - measured by STAI-S (MD = –7.18, 95% CI; –8.61, –5.76, *p* < 0.01) (3 RCTs, *n* = 274) 4. Significantly reduced depression (MD = –2.84, 95% CI: –4.09, –1.59, *p* < 0.01) (3 RCTs, *n* = 177)
Dun et al. (17) 2022	CRF, QoL	Cognitive training-related measures, social support, or a combination of both Comparator: conventional care or conventional healthy education	11 RCTs Search date: August 2021 Meta-analysis: Yes	PwCRC who had elective colorectal surgery and chemotherapy (*n* = 980)	CRF < 14 weeks: - Significant improvement in CRF (SMD = –1.13, 95% CI: –1.29, –0.96, *p* < 0.001) (9 RCTs) - Subgroup of cognitive training intervention (7 RCTs); combined cognitive training and social support intervention (2 RCTs): all statistically significant difference in CRF CRF > 14 weeks: - Significant improvement in CRF (SMD = –0.56, 95% CI: –0.77, –0.36, *p* < 0.001) (6 RCTs) - 2 RCTs included combined cognitive training and social support: non-statistically significant difference (SMD = –0.94, *p* = 0.055) QoL < 14 weeks: - Significant improvement in QoL (SMD = 0.73, 95% CI: 0.52, 0.94, *p* = 0.000) (6 RCTs) - Subgroup of cognitive training intervention (4 RCTs); combined cognitive training and social support intervention (2 RCTs): all statistically significant difference in QoL QoL > 14 weeks: - Significant improvement in QoL (SMD = 0.54, 95% CI: 0.28, 0.80, *p* = 0.003) (4 RCTs) - 1 study included combination of cognitive training and social support: No difference in QoL between intervention and control group (SMD = 0.50, *p* = 0.435)
Mosher et al. (61) 2017	Psychosocial outcomes (sexual functioning, unmet supportive care needs, distress, social support, posttraumatic growth)	Psychosocial interventions: education, individual psychotherapy, cognitive-behavioural training, group interventions	14 RCTs Search date: Oct 2016 Meta-analysis: No	PwCRC of any disease stage (*n* = 2,476)	There is limited empirical support for psychosocial interventions for CRC patients 1. Only 3 RCTs showed significant intervention effects on multiple mental health outcomes 2. The remaining 8 RCTs (wide range of psychoeducational and supportive care interventions) produced few to no effects on study outcomes
Huang et al. (62) 2023	Pain, depression, anxiety, distress, QoL	Music therapy, either active or receptive music therapy Comparator: either a different relaxation method or a standard-of-care procedure	10 RCTs Search date: August 2022 Meta-analysis: No	PwCRC or gastrointestinal cancer (*n* = 1,109)	Music therapy reduced pain, anxiety, and stress and improved QoL among cancer patients, including PwCRC - 9 out of 10 RCTs (90%) showed statistically and clinically significant improvements across outcome variables - 4 out of 7 RCTs (57%) demonstrated that music therapy reduced pain - 3 out of 4 RCTs (75%) showed that music therapy reduced anxiety
Zhang et al. (20) 2023	QoL, psychological well-being (anxiety, depression, SE, psychological distress, or related indicators)	Psychoeducation intervention Comparator: no treatment, only usual care	11 RCTs and 1 controlled clinical trial Search date: Aug 2022 Meta-analysis: Yes	PwCRC who were above 18 years of age (*n* = 1,526)	1. QoL: - Significantly improved short-term QoL (SMD = 0.30; 95% CI: 0.08, 0.52; *p* = 0.007) - No significant effect on long-term QoL ( > = 2 months) (SMD = 0.19; 95% CI: –0.02, 0.41, *p* = 0.08) (7 RCTs, *n* = 323) 2. Anxiety: - Substantial short-term (SMD = –0.73; 95% CI: – 0.94, –0.51); *p* < 0.00001) and long-term (SMD = –0.62; 95% CI: –0.87, –0.37, *p* < 0.00001) impacts on anxiety (6 RCTs, *n* = 190) 3. Depression: - Significantly reduced long-term depression (SMD = –0.37; 95% CI: –0.64, –0.11, *p* = 0.005) (3 RCTs, *n* = 229) and short-term depression (SMD = –0.55, 95% CI: –0.77, –0.34, *p* < 0.00001) (5 RCTs, *n* = 359) 4. SE: Significantly enhanced short-term SE (SMD = 0.71; 95% CI: 0.41, 1.02, *p* < 0.00001) (3 RCTs *n* = 175) 5. Psychological distress: No significant effect in either the short term (SMD = – 0.25, 95% CI: –0.65, 0.14, *p* = 0.21) or long term (SMD = –0.34, 95% CI: –0.80, 0.11, *p* = 0.14)
**Lifestyle and multimodal interventions**					
Aubrey et al. (63) 2019	Change in anthropometry, overall survival, QoL, modified habitual diet habits, fatigue, treatment compliance	Healthy eating interventions: dietary modification in habitual diet of macronutrients including fat, carbohydrate, protein, alcohol or vitamins and minerals Comparator: usual care	6 RCTs and 1 RCT protocol Search date: January 2019 Meta-analysis: Yes	Mixed cancer population including PwCRC (*n* = 2,233, PwCRC = 1,010)	1. Anthropometrics: - Weight: No significant difference between groups (2 RCTs) - BMI: reduction at 6, 12 months, & after 12 months (MD = –0.57, 95% CI: –1.07, –0.08, *p* = 0.02) (2 RCTs) - WHR: reduction in favouring the intervention group 2. Dietary components: - Reduced fat intake at 12 months (control –1.07 g versus intervention –3.06 g, *p* = 0.002) (2 RCTs); only 1 RCT shows increase in dietary fibre at 12 months; no difference for fruit and vegetable consumption (3 RCTs) 3. QoL: - 1 RCT reported an increase in QoL at 3 months in the intervention group; no difference reported in 2 RCTs - Improvements in physical QoL at 12 months after intervention (MD = 1.97, 95% CI: 0.44, 3.51, *p* = 0.01), but no difference at 6 months (MD = 0.0, 95% CI: –1.66, 1.66) (2 RCTs) - Mental domain of QoL: no effect. 4. Fatigue: no difference between groups (1 RCT) 5. Compliance: 90% attendance at face-to-face interviews, 43.5% attendance at quarterly group meetings, 72.4% successful contact with fortnightly phone calls
Moug et al. (64) 2017	Feasibility of lifestyle interventions and short and long-term health outcomes (QoL or survival)	Modification of 1 or more lifestyle factors (weight, diet, physical activity (PA), smoking and alcohol) Comparator: usual care	14 RCTs Search date: June 2015 Meta-analysis: No	PwCRC – non-metastatic (*n* = 4,674)	1. PA intervention (14 RCTs): - Significantly improved cardiorespiratory function post-intervention (1 RCT) - Significantly improved re-operative rates and earlier recovery to baseline function 2–4 months after undergoing surgery (1 RCT) - Increased 6MWT between groups pre-surgery or post-surgery (2 RCTs) - Significantly improved QoL scores after home-based telephone-guided PA intervention (1 RCT) - No cancer-free or overall survival data 3. Multimodal including dietary intervention (3 RCTs) - individualized nutrition counselling using regular foods provides most evidence in reducing radiotherapy toxicity, improving nutritional status, QoL, and improving both disease-free survival and disease-specific survival (1 RCT) - Significantly reduced BMI after 6 and 12 months and a reduction in fat intake up to 2 years (2 RCTs)
Zhou et al. (65) 2022	PA level, QoL, mental health outcomes, anthropometry	At least 1 lifestyle change (BMI, diet, PA, smoking, alcohol consumption) Comparator: usual/routine care. Placebo or no intervention	12 RCTs Search date: 1 Nov 2021 Meta-analysis: Yes	PwCRC (non-metastatic) who had completed treatment (*n* = 1,380)	1. PA level: - Significantly increased PA time after lifestyle interventions (WMD = 9.84, 95% CI, 1.20, 18.48; *p* = 0.026) (7 RCTs) - Significant increase in PA time was observed at follow-up > 6 months (WMD, 4.39; 95% CI, 0.24–8.54. *p* = 0.038) (3 RCTs); not at < 6 months (WMD = 19.17; 95% CI: 1.95, 40.29; *p* = 0.075) (4RCTs) - Significantly increased MET levels (WMD = 10.40, 95% CI: 5.30, 15.49; *p* < 0.001); no significant changes when < 6 months’ follow-up (WMD = 19.17, 95% CI, −1.95 to 40.29; *p* = 0.075) (4 RCTs) 2. QoL: - Higher FACT-C scores & improved QoL (WMD = 3.12; 95% CI: 0.24, 5. 99; *p* = 0.034) (6 RCTs) - No significant effect on fatigue: (WMD = 0.79, 95% CI: –0.59, 2.18; *p* = 0.261) (6 RCTs) 3. Mental health outcomes: - No significant effect on reducing depression (SMD = –0.16, 95% CI: –0.36, 0.05; *p* = 0.138) (4 RCTs) - No significant effect on reducing anxiety (SMD = –0.05, 95% CI: –0.28, 0.17; *p* = 0.633) (3 RCTs) 4. Anthropometric data: no effect on reducing waist circumference (WMD = 0.75, 95% CI, –2.11, 3.61, *p* = 0.608), or BMI (WMD = 0.04; 95% CI: –1.11, 1.20; *p* = 0.943) (3 RCTs)
Meng et al. (66) 2021	Primary outcome: QoL scores Secondary outcomes: anxiety and depression scores	Non-pharmacological therapies (exercise and psychotherapy, delivered by trained personnel, such as nurses, therapists, or exercise instructors) Comparator: usual care	20 RCTs Search date: 1 Aug 2020 Meta-analysis: Yes	CRC survivors of any age (*n* = 3,438)	Primary outcome: - Significant improvement in the QoL scores at 1–4 months’ follow-up (SMD = 0.368, 95% CI, 0.070, 0.665, *p* = 0.000) - No effect at 5–8 months’ follow-up (11 RCTs) Secondary outcomes: 1. Anxiety - No effect for reduced anxiety at 1–4 months’ follow-up (SMD = –0.402, 95% CI: – 0. 913, 0.109) (7 RCTs, *n* = 862) - Significant reduction in anxiety at 5–8 months’ follow-up (SMD = –0.157; 95CI: –0.312, –0.002) (5 RCTs, *n* = 644) 2. Depression - No effect for reduced depression scores at 1–4 months’ follow-up (SMD = – 0.061, 95% CI, –0.293, 0.170) (5RCTs, *n* = 619) - Significant reduction in depression at 5–8 months’ follow-up (SMD = –0.207; 95% CI: –0.390, –0.024) (4 RCTs, *n* = 465)
Molenaar et al. (67) 2022	Primary outcomes: FC (6MWT), postoperative complication rate, HRQoL Secondary outcomes: 6MWT pre-surgery, compliances, ED visit within 30 days postop, readmission within 30 days postop	Multimodal prehabilitation (moderate-intensity exercise, nutritional, and mental health support, in-hospital, outpatient, or home-based interventions) Comparator: standard routine care	3 RCTs Search date: Jan 2021 Meta-analysis: Yes	PwCRC undergoing CRC surgery (*n* = 250)	Primary outcomes: 1. FC - Improved 6MWT at 4 weeks postop but results were not statistically significant (MD = 26.02, 95% CI: –13.81, 65.85; *p* = 0.20; low CoE) (2 RCTs, *n* = 131) - Significantly improved 6MWT at 8 weeks postop (MD = 26.58,95% CI –8.88 to 62.04; *p* = 0.14; very low CoE) in favour of the prehab group (2 RCTs, *n* = 140) 2. Postop complication rate within 30 days: no significant difference (RR = 0.95,95% CI: 0.70 to 1.29; *p* = 0.75; low CoE) (3 RCTs, *n* = 250) 3. Patient-reported HRQoL: unable to perform meta-analysis. Secondary outcomes: 1. 6MWT pre-surgery, after completion of the prehabilitation programme: - Significantly improved 6MWT in favour of prehabilitation (MD = 24.91, 95% CI: 11.24, 38.57; *p* = 0.0004; moderate CoE) (3 RCTs, *n* = 225) 2. Compliance: 68–80% with exercise programme. Up to 100% in nutritional programme. 3. ED presentation within 30 days: - Reduced presentation in prehabilitation group but results were not statistically significant (RR = 0.72; 95% CI: 0.39, 1.32; *p* = 0.28, low COE) (3 RCTs, *n* = 250) 4. Readmission rates within 30 days: No effect (RR = 1.20, 95% CI: 0.54, 2.65, *p* = 0.65) (3 RCTs, *n* = 250)
Lau et al. (68) 2019	Primary outcomes: 6MWT, postoperative complications Secondary outcomes: major complications rates, SSI, pneumonia, LOS, 30-day readmission, mortality	Prehabilitation programmes (with one or more interventions including exercise, nutritional supplement, and psychological interventions) Comparator: usual care	11 RCTs (*n*(CRC) = 5 RCTs) Meta-analysis: Yes	Patients with GI cancer who are undergoing surgery (*n* = 929, *n*(CRC) = 340)	Mixed GI cancer: 1. Significant improvements in 6MWD immediately prior to surgery (MD = 32.542; 95% CI: 10.774, 54.310; *p* = 0.003) (4 RCTs) and 4–8 weeks after surgery (MD = 48.220; 95% CI: 1.532, 94.908; *p* = 0.043) (3 RCTs) 2. No significant effect for postoperative complications, major complications, SSI, pneumonia, 30-day readmission, and mortality, as well as LOS (*p* > 0.05) CRC-specific data: - No effect on reducing total postoperative complications (RR = 0.956; 95% CI: 0.616–1.483; *p* = 0.841) (5 RCTs) - No effects in major complications (RR = 0.883; 95% CI: 0.305–2.550; *p* = 0.818) (5 RCTs) - No effects on LOS (MD = 0.006; 95% CI: − 0.480 to 0.492; *p* = 0.981)
Falz et al (69) 2022	FC; postoperative outcomes (all complications, LOS)	Prehabilitation (either unimodal or multimodal, supervised, or home-based) or preoperative exercise intervention Comparator: usual care	23 mixed studies but RCT only in meta-analysis Search date: January 2022 Meta-analysis: Yes (14 RCTs)	PwCRC with scheduled CRC surgery (*n* = 1,648)	1. FC - Significant improvement in preoperative FC as measured with 6MWT (MD = 30.8, 95% CI: 13.3, 48.3; *p* = 0.0005) after prehabilitation (10 RCTs, *n* = 635) 2. LOS: - No significant effect in reducing LOS (MD = –0.26; 95% CI: –0.89, 0.37; *p* = 0.42) (12 RCTs, *n* = 1,393) 3. Postoperative overall complications - No significant effect in reducing postop complications (OR = 0.84; 95% CI 0.53, 1.31; *p* = 0.44) (11 RCTs, *n* = 1,371) - Subgroup analysis: Prehabilitation interventions lasting > 3 weeks trended towards lower overall complications (OR = 0.66; 95% CI: 0.4, 1.1; *p* = 0.11) (8 RCTs, *n* = 641); no effect if < 3 weeks (OR = 1.44, 95% CI: 0.78, 2.67, *p* = 0.24) (3RCTs, *n* = 507)
Zhang et al. (78) 2024	Primary outcomes: 6MWT Secondary outcomes: QoL, postoperative complications, total hospital LOS, postoperative LOS, healthcare expenditure	Prehabilitation or preoperative lifestyle intervention of any type, including any one of or any combination of nutritional intervention, physical training, and psychological support Comparator: routine care	28 RCTs Search date: 15 Dec 2022 Meta-analysis: Yes	PwCRC undergoing surgery (*n* = not available)	Primary outcomes: 1. Prehabilitation and/or preoperative lifestyle management significantly enhanced 6MWT compared with routine care (SMD = 1.30, 95% CI: 0.30, 2.29, *p* = < 0.01) (7 RCTs, *n* = 560) Secondary outcomes: 1. Prehabilitation or preoperative lifestyle modifications reduced postoperative complications (OR = 0.53, 95% CI: 0.40, 0.69; *p* < 0.01) (22 RCTs, *n* = 2,989) 2. No significant effect for QoL (SMD = 1.81, 95% CI: −0.26, 3.87) (4 RCTs, *n* = 278) 3. No significant effect for postoperative LOS (SMD = −1.46, 95% CI: −3.12, 0.20) (10 RCTs, *n* = 1,823) or total hospital LOS (SMD = −0.26, 95% CI: −0.68, 0.15) (8 RCTs, *n* = 1,136) 4. Insufficient data on health expenditure
Zhou et al. (79) 2024	6MWT, postoperative complications, LOS, and anxiety and depression scores (HADS)	Multimodal prehabilitation or exercise prehabilitation Comparator: no treatment or enhanced recovery pathway	17 RCTs Search date: 19 Dec 2023 Meta-analysis: Yes	PwCRC undergoing surgery (*n* = 1,961)	1. FC: - Exercise and multimodal prehabilitation improves 6MWT (MD = 29.00, 95% CI: 26.64, 31.36; *p* < 0.001) (11 RCT, *n* = 886) - Exercise prehabilitation alone does not effectively improve 6MWT (MD = 13.24; 95% CI: −37.89, 64.36; *p* = 0.61) (3 RCTs, *n* = 82) - Multimodal prehabilitation improves 6MWT (MD = 29.03; 95% CI: 26.67, 31.39; *p* < 0.001) - Multimodal subgroup analysis based on duration of follow up: 6MWT improved before surgery (MD = 34.77; 95% CI: 16.76, 52.7; *p* = 0.0002), but did not improve 4 weeks (MD = 10.17; 95% CI: –9.82, 30.16, *p* = 0.32) and 6 to 8 weeks after surgery (MD = 17.78; 95% CI: −20.77, 56.33; *p* = 0.37) 2. LOS: - No reduction in LOS (MD = –0.14; 95% CI: –0.31, 0.03; *p* = 0.1) (13 RCTs, *n* = 1,730) 3. Postoperative complications: - No reduction due to either intervention (RR = 0.87; 95% CI: 0.71, 1.07; *p* = 0.20) (11 RCTs, *n* = 1,387) - Multimodal prehabilitation subgroup (RR = 0.78; 95% CI: 0.65, 0.94; *p* = 0.01) effectively reduced postoperative complications (7 RCTs, *n* = 601) 4. Anxiety and depression scores: - Both exercise prehabilitation and multimodal prehabilitation improves anxiety score (MD = −0.71; 95% CI: −1.41, −0.01; p = 0.05) (5 RCTs, *n* = 382) - No effect on the interventions in terms of depression score reduction (SMD = −0.10; 95% CI: −0.30, 0.09; *p* = 0.29) (6 RCTs, *n* = 419)

ACT: acceptance and commitment therapy; ALB: albumin; ARP: anal resting pressure; BDI: Beck Depression Index; BMI: body mass index; BFI: Brief Fatigue Inventory; CBI-B: Cancer Behaviour Inventory-Brief form; CI: conference interval; CrI: credible interval; COE: classes of evidence; CBT: cognitive behavioural therapy; CRF: cancer related fatigue; CRP: C-reactive protein; EA: electroacupuncture; ECOG: Eastern Cooperative Oncology Group Scale; ED: Emergency Department; EIN: enteral immunonutrition; ERAS: Enhanced Recovery After Surgery; EORTC QLQ-CR29: European Organization for Research and Treatment of Cancer Quality of Life Questionnaire-Core 29; EORTC QLQ-CR30: European Organization for Research and Treatment of Cancer Quality of Life Questionnaire-Core 30; FACT-C: Functional Assessment of Cancer Therapy-Colorectal scale; FACT-F: Functional Assessment of Cancer Therapy-Fatigue scale; FACT-G: Functional Assessment of Cancer Therapy-General scale; FACIT-F: Functional Assessment of Chronic Illness Therapy-Fatigue score; FC: functional capacity; FA: fatty acid; FI: faecal incontinence; FSI: Fatigue Symptom Inventory; GI: gastrointestinal; Gln: glutamine; GSE: General Self-Efficacy scale; GRADE: Grade Of Recommendations, Assessments, Development and Evaluation Working Group Grades of Evidence; HADS: Hospital Anxiety and Depression Scale; HRQoL: Health-related Quality of Life; hsCRP: high sensitivity C-reactive protein; IL: interleukin; IL-6: interleukin-6; IMN: iImmunonutrition; LARS: LOW ANTERIOR RESECTION SYNDROME; LOS: length of stay; MA: manual acupuncture; MET: metabolic equivalent task; MFI: Multidimensional Fatigue Inventory; MD: mean difference; MSP: maximum squeeze pressure; NB: nitrogen balance; NIH PROMIS: National Institutes of Health Patient Reported Outcomes Measure Information System; N-3 PUFA: N-3 polyunsaturated fatty acids; OR: odds ratio; ONS: oral nutritional supplements; PA: physical activity; PFMT: pelvic floor muscle training; PIN: parenteral immunonutrition; PONV: postoperative nausea/vomiting; QoL: quality of life; RFPS: Revised Piper Fatigue Scale; RRP: rectal resting pressure**;** SMD: standardized mean difference; SSES: Stoma Self-Efficacy Scale; STAI: State-Trait Anxiety Inventory; SUCRA: surface under the cumulative ranking curve; SF-12: Short Form Health Survey; SSI: surgical site infection; sTCAM-1: soluble intercellular adhesion molecule-1; TNF-a: tumor necrosis factor-alpha; TOI: Trial Outcome Index; TP: total protein; TUG: Timed Up & Go test; UC: usual care; UTI: urinary tract infection; VAS: visual analogue scale; VO2Max: peak oxygen uptake; WMD: weighted mean difference; 6MWT: 6 Min Walk Test.

### Methodological quality of included studies

Overall, only 4 reviews including 2 Cochrane reviews were assessed as “high quality” (18, 53, 66, 67), 27 reviews were of “moderate quality” (13–15, 19, 20, 30, 34, 36–38, 40, 42, 45, 47–49, 52, 54, 56, 58, 59, 65, 70, 72, 74, 77, 79), 15 reviews of “low quality” (16, 32, 33, 41, 43, 46, 57, 61, 62, 68, 69, 71, 73, 76, 78) and 14 reviews of “critically low quality” (12, 17, 29, 31, 35, 39, 44, 46, 50, 55, 60, 63, 64, 75) (Table II).

**Table II:** AMSTAR2 assessment

AMSTAR2 items Author, year	1	2	3	4	5	6	7	8	9	10	11	12	13	14	15	16	Overall score
Da Silva Bezerra et al. (29) 2021	N	N	N	PY	YY	YY	N	N	YY	N	NA	NA	N	N	NA	YY	Clow
Cramer et al. (30) 2013	YY	YY	N	PY	YY	YY	N	YY	YY	N	YY	YY	YY	YY	NA	YY	Moderate
Jung et al. (31) 2021	YY	PY	N	PY	YY	N	N	YY	YY	N	N	N	N	N	N	YY	Clow
Gao et al. (16) 2020	YY	YY	N	PY	YY	YY	N	YY	YY	N	YY	N	N	N	NA	YY	Low
Dun et al. (32) 2020	N	PY	N	PY	N	YY	N	PY	YY	N	YY	N	N	YY	YY	YY	Low
Geng et al. (33) 2023	YY	PY	N	PY	YY	YY	N	YY	YY	YY	YY	YY	YY	YY	N	YY	Low
Wang et al. (34) 2022	YY	PY	N	PY	N	YY	N	N	PY	N	YY	YY	YY	YY	NA	YY	Moderate
Kraemer et al. (35) 2022	YY	PY	N	PY	YY	YY	N	YY	PY	N	N	N	N	N	N	YY	Clow
Singh et al. (36) 2020	YY	YY	N	PY	N	N	N	N	PY	N	YY	YY	YY	YY	YY	YY	Moderate
Brandenbarg et al. (37) 2018	YY	YY	N	PY	YY	YY	N	YY	YY	N	YY	YY	YY	YY	YY	YY	Moderate
Lund et al. (15) 2020	YY	YY	N	PY	YY	N	N	YY	YY	N	YY	YY	YY	YY	YY	YY	Moderate
McGettigan et al. (18) 2020	YY	YY	N	YY	YY	YY	N	YY	YY	YY	YY	YY	YY	YY	NA	YY	High
Abdul Razak et al. (70) 2024	YY	YY	N	PY	YY	YY	N	YY	YY	N	YY	N	YY	YY	NA	YY	Moderate
Su et al. (71) 2024	YY	N	N	Y	Y	Y	N	Y	Y	N	Y	N	YY	N	NA	YY	Low
Nakashima et al. (72) 2023	YY	YY	N	YY	YY	YY	YY	PY	YY	N	YY	YY	YY	N	YY	YY	Moderate
Pun et al. (73) 2024	YY	YY	N	PY	YY	YY	N	YY	YY	N	YY	YY	YY	YY	N	YY	Low
Liu et al. (38) 2016	YY	PY	N	PY	N	YY	N	YY	YY	N	YY	YY	YY	YY	NA	YY	Moderate
Wierzbicka et al. (39) 2021	N	PY	N	PY	YY	YY	N	N	YY	N	NA	NA	N	N	N	YY	Clow
Dikeocha et al. (40) 2022	YY	PY	N	PY	YY	YY	N	PY	YY	N	NA	NA	YY	N	YY	YY	Moderate
Amitay et al. (41) 2020	YY	YY	N	PY	YY	YY	N	YY	YY	YY	YY	N	N	YY	NA	YY	Low
Khan et al. (42) 2023	YY	PY	N	PY	YY	N	N	N	YY	N	YY	YY	YY	N	YY	YY	Moderate
Shen et al. (14) 2022	YY	YY	N	PY	YY	YY	N	PY	YY	N	YY	YY	YY	N	YY	YY	Moderate
Bruns et al. (43) 2018	YY	YY	YY	PY	YY	YY	N	YY	YY	N	YY	YY	YY	N	N	YY	Low
Xu et al. (44) 2018	N	YY	N	PY	N	N	N	N	YY	N	YY	N	N	YY	N	N	Clow
Jiang et al. (12) 2020	YY	PY	N	PY	N	YY	N	YY	YY	N	YY	N	N	N	N	YY	Clow
Yue at al. (45) 2022	YY	YY	N	PY	YY	YY	N	YY	YY	N	YY	YY	YY	YY	YY	YY	Moderate
Li et al. (46) 2023	YY	YY	N	PY	YY	YY	N	YY	YY	N	YY	YY	YY	YY	N	YY	Low
Ye et al. (47) 2023	YY	PY	N	PY	YY	YY	N	PY	YY	N	YY	YY	YY	N	YY	YY	Moderate
Veziant et al. (48) 2023	YY	YY	N	PY	YY	YY	N	PY	PY	N	YY	YY	YY	YY	YY	YY	Moderate
Yang et al. (49) 2021	YY	YY	N	PY	YY	YY	N	PY	YY	N	YY	YY	YY	YY	YY	YY	Moderate
Jolfaie et al. (50) 2015	N	PY	N	PY	YY	N	N	PY	PY	N	NA	NA	N	N	N	YY	Clow
Liu et al. (51) 2023	N	YY	N	PY	YY	YY	N	PY	YY	N	N	N	N	N	YY	YY	Clow
Chen et al. (52) 2022	YY	YY	N	PY	N	YY	N	PY	YY	N	YY	YY	YY	YY	YY	YY	Moderate
An et al. (53) 2022	YY	YY	N	YY	YY	N	YY	YY	YY	N	YY	YY	YY	YY	NA	YY	High
Probst et al. (13) 2017	N	YY	N	PY	YY	YY	N	N	YY	YY	YY	YY	YY	YY	YY	YY	Moderate
Xiong et al. (74) 2023	YY	YY	N	PY	YY	YY	N	N	YY	N	YY	N	YY	YY	YY	YY	Moderate
Qin et al. (75) 2024	YY	N	N	PY	YY	YY	N	PY	YY	N	YY	YY	YY	YY	N	YY	Clow
Persson et al. (76) 2024	N	YY	N	PY	YY	YY	N	N	YY	N	YY	YY	YY	YY	YY	N	Low
Zhang et al. (77) 2024	YY	YY	N	YY	YY	YY	N	N	YY	N	YY	YY	YY	YY	YY	YY	Moderate
Liu et al. (54) 2018	YY	PY	N	PY	YY	YY	N	PY	YY	N	YY	YY	YY	YY	YY	YY	Moderate
Kim et al. (55) 2016	YY	YY	N	PY	YY	YY	N	N	YY	N	N	N	N	N	NA	YY	Clow
Liu et al. (56) 2017	YY	YY	N	PY	YY	YY	N	PY	YY	N	YY	YY	YY	YY	NA	YY	Moderate
Xu et al. (57) 2021	YY	PY	N	PY	YY	YY	N	PY	YY	N	YY	YY	YY	N	N	YY	Low
Qi et al. (58) 2023	YY	YY	N	PY	YY	YY	N	YY	YY	YY	YY	YY	YY	YY	NA	YY	Moderate
Zhao et al. (59) 2023	YY	YY	N	PY	YY	YY	N	PY	YY	N	YY	YY	YY	YY	YY	YY	Moderate
Son et al. (19) 2019	YY	PY	N	PY	YY	YY	N	PY	YY	N	YY	YY	YY	N	YY	YY	Moderate
Wan et al. (60) 2022	YY	YY	N	PY	YY	YY	N	YY	YY	N	N	YY	YY	N	N	YY	Clow
Dun et al. (17) 2022	YY	YY	N	PY	YY	YY	N	PY	PY	N	N	YY	N	N	YY	YY	Clow
Mosher et al. (61) 2017	N	PY	N	PY	YY	YY	N	YY	YY	N	NA	NA	YY	N	N	YY	Low
Huang et al. (62) 2023	N	PY	N	PY	YY	YY	N	N	YY	N	NA	NA	YY	N	N	YY	Low
Zhang et al. (20) 2023	YY	YY	N	PY	YY	YY	N	YY	YY	N	YY	N	YY	YY	YY	YY	Moderate
Aubrey et al. (63) 2019	YY	PY	N	YY	N	YY	N	YY	PY	N	YY	N	N	YY	N	YY	Clow
Moug et al. (64) 2017	N	PY	N	PY	YY	YY	N	N	PY	N	NA	NA	N	YY	N	YY	Clow
Zhou et al. (65) 2022	YY	YY	N	PY	YY	YY	N	YY	YY	N	YY	N	YY	YY	YY	YY	Moderate
Meng et al. (66) 2021	YY	YY	YY	PY	YY	YY	N	YY	PY	N	YY	YY	YY	YY	YY	YY	High
Molenaar et al. (67) 2022	YY	YY	YY	YY	YY	YY	YY	YY	YY	YY	YY	YY	YY	YY	NA	YY	High
Zhang et al. (78) 2024	YY	YY	N	PY	YY	YY	N	N	YY	N	YY	N	N	YY	YY	YY	Low
Zhou et al. (79) 2024	YY	YY	N	PY	YY	YY	N	PY	YY	N	YY	YY	YY	YY	YY	YY	Moderate
Lau et al. (68) 2019	N	N	N	PY	N	N	N	N	PY	N	YY	YY	YY	N	YY	N	Low
Falz et al. (69) 2022	YY	YY	N	PY	YY	YY	N	YY	YY	N	YY	N	N	YY	YY	YY	Low

AMSTAR2: A MeaSurement Tool to Assess systematic Reviews 2 appraisal tool; YY: yes; PY: partial yes; N: no; NA: not applicable; Clow: critically low.

The majority of reviews used the Patient Intervention Control Outcome (PICO) description as an organizing framework in the inclusion criteria, except for 9 reviews (32, 39, 44, 50, 51, 61, 62, 68, 76). Most reviews provided an explicit statement on registered information of the protocol prior to the conduct of the review, except for 4 reviews (29, 68, 71, 75). Only 2 reviews provided an explanation of their selection of the study designs for inclusion (43, 67). All reviews searched at least 2 databases, justified publication restrictions, and provided keywords or search strategies. Thirteen reviews did not perform screening or data extraction in duplicate (12, 15, 31, 32, 34, 36, 38, 42, 44, 50, 52, 53, 68). Only 3 reviews provided a list of excluded studies and justified the exclusions (18, 53, 67). Fifteen reviews did not adequately describe the PICO details and research designs of included primary studies (13, 29, 34, 36, 39, 42, 44, 55, 62, 64, 68, 74, 76–78). All studies assessed the quality of the primary studies using validated risk of bias (RoB) tools. The majority of reviews, except for 6 (17, 31, 35, 51, 55, 60) conducted meta-analysis using appropriate methods for statistical combination of results. Seventeen reviews did not assess the potential impact of RoB in individual studies on the results of the meta-analysis (12, 16, 20, 31, 32, 35, 41, 44, 46, 55, 63, 65, 69–71, 74, 77). Twenty-nine reviews provided satisfactory explanations and discussions of any heterogeneity observed (12, 14, 16, 17, 19, 29, 31, 35, 39, 40, 42, 43, 47, 50, 51, 55, 57, 60–62, 68, 70, 73–79). Twenty-eight reviews carried out adequate investigation of publication bias (13–15, 17, 19, 20, 32, 36, 37, 40, 42, 45, 47–49, 51, 52, 59, 65, 66, 68, 69, 72, 74, 76–79), while 13 reviews did not apply funnel plots due to the low number of included primary studies (16, 18, 30, 34, 38, 41, 53, 55, 56, 58, 67, 70, 71). Only 6 reviews reported on sources of funding for primary studies (13, 18, 33, 41, 58, 67). The majority of the reviews, except for 3 (44, 68, 76), declared potential sources of conflict of interest.

### Evidence synthesis of rehabilitation interventions

Of the 60 included reviews, 16 evaluated the effect of exercise interventions (15, 16, 18, 29–37, 70–73); 23 reviews evaluated the effects of nutritional interventions including pro/synbiotics and a range of immunonutrition programmes (12–14, 38–53, 74–77) and 6 reviews examined complementary and alternative medicine (CAM) interventions (acupuncture) (54–59). The effects of lifestyle interventions and multimodal rehabilitation programmes were studied in 9 reviews (63–69, 78, 79). The effects of psychosocial interventions were evaluated in 6 reviews (17, 19, 20, 60–62). There was marked heterogeneity of the evaluated interventions and measured outcomes among the included reviews. The characteristics and outcomes of these interventions are summarized in Table I and best evidence for commonly studied outcomes based on the type of intervention using the GRADE approach in Table III.

**Table III:** GRADE assessment of included studies

Intervention	Outcomes	Number of studies and participants	Point estimates	Interval overlaps	Direction of effect	Magnitude of hetero-geneity	CI not consis-tent	Sample size magni-tude	Magni-tude of number of studies	Outcome as common event	Harm from treat-ment	Overall GRADE
Exercise intervention	Improved physical fitness	7 SRs, 42 RCTs, *n* = 1,951	Yes	Substantial overlap	Yes	Low– Moderate	No	High	Large	Yes	No	Moderate
	Improved QoL	12 SRs, 59 RCTs, *n* = 4354	Yes	Some overlap	Yes	Moderate	No	High	Large	Yes	No	Moderate
	Reduced CRF	12 SRs, 64 RCTs, *n* = 4,651	No	Some overlap	Yes	Moderate	No	High	Large	Yes	No	Low
	Improved disease-related mental health: depressive/ anxiety symptoms	Depression: 6 SRs, 17 RCTs, *n* = 1,852 Anxiety: 6 SRs, 15 RCTs, *n* = 1,534	Yes	Substantial overlap	Yes	Mostly Low	No	High	Large	Yes	No	Very Low
	Improved anthropometry measures	BMI: 3 SRs, 16 RCTs, *n* = 1,082 % body fat: 2 SRs, 11 RCTs, *n* = 636	Yes	Some overlap	Yes	Moderate	No	High	Moderate–Large	Yes	No	Low
Nutritional intervention	Improved humoral immunity	Total: 9 SRs, 84 RCTs, *n* = NA	Yes	Substantial overlap	Yes	Low–Moderate	No	High	Large	Yes	No	Moderate
	Improved adaptive immunity		Yes	Substantial overlap	Yes	Moderate	Yes	High	Large	Yes	No	Low
	Improved serum inflammatory markers		Not comparable	Not comparable	Yes	Moderate–High	No	High	Large	Yes	No	Moderate
	Reduced infection complication rates	Total: 15 SRs, 125 RCTs, *n* = incomplete data	Yes	Substantial overlap	Yes	Mostly Low	No	High	Large	Yes	No	High
	Reduced non-infectious complications/ anastomotic leak	Total: 6 SRs, 45 RCTs, *n* = NA	No	Some overlap	Yes	Low	Yes	High	Large	Yes	No	Low
	Reduced mortality	Total: 3 SRs, 17 RCTs, n = NA	No	Some overlap	No	Low	No	High	Large	Yes	No	Very Low
	Reduced hospital LOS	Total: 12 SRs, 79 RCTs, n = NA	Yes	Some overlap	Yes	Half are High, half are Low–Moderate	No	High	Large	Yes	No	Moderate
Acupuncture intervention	Improved GI functional recovery	Total: 5 SRs, 51 RCTs, *n* = 3,776	Yes	Substantial overlap	Yes	Mostly High	No	High	Large	Yes	No	Moderate
	Reduced postoperative pain	Total: 3 SRs, 13 RCTs, *n* = 1,873	No	Not comparable	No	Low	Yes	High	Large	Yes	No	Very Low
	Reduced LOS	Total: 2 SRs, 9 RCTs, *n* = 711	Yes	Some overlap	No	Low	No	High	Moderate	Yes	No	Low
Psychosocial intervention	Improved QoL	Total: 4 SRs, 25 RCTs, *n* = NA	Yes	Some Overlap	Yes	Low-Moderate	No	High	Large	Yes	No	Moderate
	Improved SE	Total: 2 SRs, 10 RCTs, *n* = NA	Not comparable	Not comparable	No	Moderate	Yes	High	Moderate	Yes	No	Very Low
	Reduced anxiety	Total: 2 SRs, 8 RCTs, *n* = 633	Not comparable	Not comparable	Yes	Low	No	High	Moderate	Yes	No	Moderate
	Reduced depression	Total: 2 SRs, 8 RCTs, *n* = 536	Not comparable	Not comparable	Yes	Low–Moderate	Yes	High	Large	Yes	No	Moderate
Multimodal prehabilitation intervention	Improved preoperative FC	Total: 5 SRs, 35 RCTs, *n* = 2,540	Yes	Substantial overlap	Yes	Moderate	No	High	Large	Yes	No	High
	Reduced hospital LOS	Total: 4 SRs, 33 RCTs, *n* = NA	Yes	Some overlap	No	Low	No	High	Large	Yes	No	Very Low
	Reduced postoperative complications	Total: 5 SRs, 45 RCTs, *n* = NA	Yes	Some overlap	Yes	Low–Moderate	No	High	Large	Yes	No	Low
Lifestyle intervention: at least 1 lifestyle change (diet/healthy eating, BMI, PA, smoking, and alcohol consumption)	Improved QOL	Total: 3 SRs, 18 RCTs, *n* = NA	Not comparable	Not comparable	Yes	Moderate–High	No	High	Large	Yes	Yes	Moderate
	Reduced depression	Total: 2 SRs, 8 RCTs, *n* = 851	Yes	Some overlap	Yes	Low	Yes	High	Moderate	Yes	No	Low
	Reduced anxiety	Total: 2 SRs, 10 RCTs, *n* = 1,190	Yes	Some overlap	Yes	Low	Yes	High	Moderate	Yes	No	Low
	Improved anthropometry	Total: 2 SRs, 5 RCTs, *n* = NA	Yes	Some overlap	No	Moderate	Yes	High	Moderate	Yes	No	Very Low

GRADE: Grade of Recommendations; Assessment: Development, and Evaluation working group grades of evidence; 6MWT: 6 Min Walk Test; BMI: body mass index; CI: conference interval; CRF: cancer-related fatigue; FC: functional capacity; HADS: Hospital Anxiety and Depression Scale; HRQoL: Health-related Quality of Life; LOS: length of stay; QoL: quality of life; SR: systematic review; VO2Max: peak oxygen uptake. NA = not available, due to data in original systematic reviews not being specified.

The specific rehabilitation interventions evaluated in the included reviews and key findings are summarized below:

*Exercise interventions*. Of the 16 reviews (*n* = 152 RCTs) that studied the effects of exercise interventions, 12 reviews included PwCRC who had completed their primary cancer treatment (16, 18, 29–32, 34, 37, 70–73), while 3 reviews included all patients regardless of treatment status (33, 35, 36), and 1 review included patients undergoing chemotherapy treatment (15). Exercise interventions incorporated various modalities including aerobic exercises, resistance exercise, mixed mode exercise, or others (e.g., yoga). These programmes were of variable duration and intensity, comprising either home-based or centre-based therapy, which can be self-directed, supervised, or a combined mode of delivery. The overall findings supported the effectiveness of exercise interventions in PwCRC in improving their physical fitness, QoL, disease-related mental health, anthropometry measures, and reducing CRF. Safety data of exercise interventions remained limited and was reported in only 4 reviews (15, 18, 36, 72), which found no serious adverse events or safety concerns related to exercise interventions in comparison with usual care.

Seven reviews (15, 16, 18, 30, 31, 35, 36) (*n* = 42 RCTs) investigated the effects of exercise on physical fitness, demonstrating “moderate quality” evidence in improving patients’ physical fitness and aerobic capacity. One Cochrane systematic review (18) confirmed the positive effects of physical activity interventions in improving aerobic fitness in PwCRC (standard mean difference [SMD] = 0.82, 95% CI = 0.34 to 1.29, *p* = 0.0007); however. these beneficial effects were not sustained at 6 months (SMD = 0.44, 95% CI = –0.04 to 0.92, *p* = 0.07).

Twelve reviews (*n* = 55 RCTs) investigated the effect of exercise interventions on QoL (15, 16, 18, 29–32, 34–36, 70, 71). One descriptive review (*n* = 4 RCTs) found no significant improvement in the QoL of PwCRC post unsupervised or semi-supervised aerobic exercise interventions (29). Similar findings of non-statistically significant results were demonstrated by Gao et al. (16) (*p* = 0.06), Cramer et al. (30) (*p* = 0.08), Abdul Razak et al. (70) (*p* = 0.06), and Kraemer et al. (35) in both home-based (*p* = 0.31) and supervised exercise (*p* = 0.07) intervention groups. In contrast, 7 reviews found statistically significant improvements in QoL outcomes (disease-specific QoL, social factors of QoL, and HRQoL after exercise interventions) with large effect sizes (15, 18, 31, 32, 34, 36, 71). McGettigan et al. (18) found “moderate-quality” evidence of physical activity interventions in improving HRQoL in PwCRC at immediate-term follow up (SMD = 0.36, 95% CI 0.10 to 0.62, *p* = 0.007) and at 12 weeks to 6 months’ follow-up (SMD 0.70, 95% CI 0.14 to 1.26, *p* = 0.01). In a more recent study, Su et al. (71) reported similar improvement in HRQoL after internet-based digital health exercise interventions at 6 months (p = 0.03). Overall findings suggest “moderate-quality” evidence for the exercise interventions in improving QoL outcomes in PwCRC.

Two reviews explored the effect of pelvic floor muscle training (PFMT) on QoL with inconsistent findings (72, 73). Nakashima et al. (72) found no trend toward a positive impact on HRQoL after PFMT intervention by comparing data from 2 RCTs. On the other hand, Pun et al. (73) reported a statistically significant positive effect on QoL components. The quality of evidence for PFMT is inconclusive due to lack of data.

The efficacy of exercise interventions on CRF was investigated in 12 reviews (15, 16, 18, 30–34, 36, 37, 70, 71) (*n* = 64 RCTs), with 7 reviews demonstrating statistically significant improvements (15, 18, 32–34, 36, 71). A Cochrane review reported low-quality evidence for physical activity interventions in improving CRF in PwCRC in the immediate short term (SMD = 2.16, 95% CI = 0.18 to 4.15, *p* = 0.04) (18). Three reviews (16, 30, 37) noted a trend for reduced CRF score observed in all exercise intervention groups, but, the results did not reach the statistical significance level. Overall findings suggest “low-quality” evidence for exercise interventions in improving CRF outcomes in PwCRC.

Seven reviews (*n* = 19 RCTs) investigated the effects of exercise interventions in improving disease-related mental health outcomes (15, 16, 18, 34, 36, 70, 71). Six reviews (*n* = 17 RCTs) compared depression scores after exercise interventions (15, 16, 18, 36, 70, 71) and found no statistically significant improvements except in 1 review (36) (SMD = 0.35, 95% CI = 0.02 to 0.67, *p* = 0.04). Six reviews (*n* = 15 RCTs) evaluated anxiety scores (16, 18, 34, 36, 70, 71) and observed no statistically significant effects. Overall findings suggest exercise interventions had “Very low-quality” evidence in improving disease-related mental health outcomes in PwCRC.

Four reviews (*n* = 27 RCTs) evaluated anthropometry measures such as body mass index (BMI) or percentage body fat (16, 18, 31, 36), with 3 reviews (*n* = 16 RCTs) demonstrating limited evidence for exercise interventions in improving BMI in PwCRC (16, 18, 31). Singh et al. (36) found significantly reduced body fat in the intervention group (SMD = 0.51, 95% CI = 0.05 to 0.97, *p* = 0.03). Overall, there was “low-quality” evidence for exercise interventions for improved anthropometry measures.

*Nutritional interventions.* The effects of various nutritional interventions were assessed in 23 systematic reviews (*n* = 327 RCTs) including 20 meta-analyses (12–14, 38, 41–49, 51–53, 74–77) and 3 descriptive reviews (39, 40, 50). Interventions comprised a range of dietary supplements including macronutrients (proteins, carbohydrates, fats) and micronutrients (pro/synbiotics, immunonutrition, and vitamin supplements) administered via various routes. The immunonutrition (IMN) formula includes arginine, omega-3 polyunsaturated fatty acid (PUFA), glutamine, and nucleotides. All studies were performed in PwCRC during primary cancer treatment (chemotherapy, chemoradiotherapy, or radical CRC resection).

Two reviews (*n* = 29 RCTs) (39, 40) studied the effects of probiotics/synbiotics in PwCRC and found enhanced gut microbiota diversity with reduced pathogenic bacterial load, postoperative bacterial translocation, transmucosal permeability, and increased colon mucosal transepithelial resistance. These findings were consistent with another meta-analysis, which found significantly lower permeability (SMD = 3.83, *p* = 0.000) and higher intestinal mechanical barrier function (SMD = 4.74, *p* = 0.000) post probiotics/synbiotics (38).

Nine reviews (*n* = 84 RCTs) investigated nutritional interventions on immunity and serum inflammatory markers (38, 45–47, 49, 51, 52). Humoral immunity was measured by serum antibodies (IgA, IgM, and IgG) levels. Five reviews showed statistically significant increases in serum IgA level (38, 45, 49, 52, 77). Four reviews found statistically significant increases in serum IgM level (45, 49, 52, 77), 3 supported statistically significant increases in serum IgG level (49, 52, 77) and 1 failed to achieve statistical significance (45). Nutritional interventions including probiotics, glutamine, and Omega-3 PUFA supplements significantly increased the adaptive immunity (measured by CD4/CD8 cell ratio and CD4 cell counts) in 3 (45, 49, 52) out of 5 reviews (45, 46, 49, 52, 77). Two reviews found statistically significant reduction of CD8 cell counts after probiotics and PUFA supplements (45, 49); however, this result was not replicated in 2 other reviews (46, 52). Overall, there was “moderate-quality” evidence that IMN and pre/pro(syn)biotics improved humoral immunity and “low-quality” evidence for improved adaptive immunity. A range of serum inflammatory markers including TNF-alpha, IL-6, albumin, and CRP, were studied in 6 reviews (38, 46, 47, 51, 74, 77). Four reviews found significantly reduced serum TNF-alpha level and serum IL-6 level in the intervention group (46, 47, 51, 74). Results were inconsistent for effects on serum albumin levels in 4 reviews (38, 46, 74, 77). Six reviews investigated CRP levels following nutritional interventions (38, 46, 47, 51, 74, 77), with 4 reviews showing significant improvements (46, 51, 74, 77), while 2 demonstrated no significant effects (47, 51). Overall, there was “moderate-quality” evidence of nutritional interventions during CRC treatment for improved immune function and serum inflammatory markers.

The effects of nutritional interventions on infectious complications including surgical site infection, septicaemia, and pneumonia were studied in 15 reviews (12–14, 41–44, 46–49, 53, 74, 76, 77). Of the 13 reviews that investigated the outcome of overall infectious complications (12-14, 41, 42, 44, 46–48, 53, 74, 76, 77), 12 found statistically fewer overall infectious complications post-nutritional interventions (12-14, 41, 42, 44, 47, 48, 53, 74, 76, 77). Li et al. (46) found some improved infectious complication rates after nutritional interventions, although this was not statistically significant (risk ratio [RR] = 0.72, *p* = 0.29). Further, 8 reviews investigated the incidence of surgical site infections after nutritional interventions (14, 43, 44, 47–49, 76, 77), of which 7 reviews found statistically significant reduced surgical site infection rates. Bruns et al. (43) demonstrated that preoperative nutritional interventions as part of CRC prehabilitation reduced incision site infection rates (OR 0.57; 95% CI = 0.30 to 1.09); however, this effect failed to achieve statistical significance (*p* = 0.09) (43). Overall, there was “high-quality” evidence for nutritional interventions towards improved postoperative infectious complication rates in patients who are undergoing CRC surgery.

Six reviews evaluated the effects of nutritional interventions upon overall non-infectious complications such as intestinal obstruction, wound dehiscence, or deep vein thrombosis (12, 14, 47–49, 77). One review showed lack of evidence for enteral immunonutrition towards overall non-infectious complications (RR = 1.15, 95% CI = 0.71 to 1.87, *p* = 0.58) (14). Another review demonstrated that glutamine, PUFAs, and arginine-based immunonutrition interventions reduced overall non-infectious complications (12). Four out of 5 reviews found non-significant findings regarding the outcome of anastomotic leak (12, 47–49, 77). Overall, there was “low-quality” evidence that nutritional interventions improved overall non-infectious complication rates after CRC treatment.

Three reviews evaluating the effects of nutritional interventions on mortality risk (13, 51, 53) demonstrated non-significant effects associated with perioperative mortality. The effects of nutritional interventions upon hospital LOS in patients were evaluated in 12 reviews (12–14, 41, 44, 46, 47, 49, 51, 53, 76, 77). Seven reviews found statistically significant reduction in LOS (12, 14, 44, 47, 49, 51, 53). Li et al. (46) did not find a statistically significant reduction in overall LOS (weighted mean difference [WMD] = −1.19, 95% CI = −2.62 to 0.24, *p* = 0.10), but reported a significant LOS reduction in a subgroup analysis of both pre- and postoperative supplementation group (preoperative WMD = −2.27, 95% CI = −3.58 to −0.97, *p* < 0.001; postoperative WMD = −2.66, 95% CI = −4.70 to −0.62, *p* = 0.01). Overall, there was “moderate-quality” evidence for nutritional interventions for reduced hospital LOS in PwCRC undergoing primary treatment.

*Acupuncture interventions.* The effects of acupuncture interventions in perioperative management (surgical resection) of CRC were evaluated in 6 meta-analyses (*n* = 80 RCTs) (54–59). Acupuncture interventions included electroacupuncture (EA), manual acupuncture (MA), acupressure, moxibustion, point application, and laser acupuncture, or any combination. The overall findings supported the effectiveness of acupuncture interventions in improving postoperative gastrointestinal functional recovery, reduced postoperative nausea, pain and hospital LOS.

Five reviews (*n* = 51 RCTs) investigated the effects of acupuncture on postoperative gastrointestinal functional recovery in patients who underwent CRC surgery (54–56, 58, 59) and found “statistically significant reduced time to first bowel sound” (54, 56), reduced time to first flatus (54–56, 58, 59), and shortened time to first defecation (54–56, 58, 59). Three reviews investigated the effects of perioperative acupuncture on postoperative pain and found largely inconsistent results (55, 56, 58). Liu et al. (56) in a meta-analysis reported less opioid consumption after acupuncture treatment, while both Kim et al. (55) and Qi et al. (58) found no statistically significant benefit of acupuncture on postoperative pain management. Overall, there was “very low-quality” evidence to suggest that acupuncture interventions reduced postoperative pain.

Two reviews evaluated the effects of acupuncture interventions on LOS and found inconsistent results (56, 59). In a meta-analysis, Liu et al. (56) reported a non-superior effect of acupuncture interventions in reducing hospital LOS (SMD = −0.18, 95% CI = −0.46 to 0.10; *p* = 0.20); however, subgroup analysis showed significantly shorter LOS (SMD = −0.32, 95% CI = 0.61 to 0.03, *p* = 0.03) with EA. In another meta-analysis, Zhao et al. (59) reported statistically significantly shorter LOS in the acupuncture group (SMD = −0.40, 95% CI = −0.60 to −0.21, *p* < 0.0001). Overall, there was “low-quality” evidence for acupuncture interventions for reduced hospital LOS.

*Psychosocial interventions.* Psychosocial interventions evaluated comprised cognitive behavioural therapy (CBT), acceptance and commitment therapy (ACT), psychotherapy, psychoeducation, counselling, relaxation therapy, supportive therapy, motivational interviewing, friendship association, group discussions, and structured family therapy. These interventions were delivered via face-to-face, telehealth, or web-based (mobile applications, websites, online portals, or forums). Six systematic reviews (*n* = 66 RCTs) (17, 19, 20, 60–62) assessed the effects of psychosocial interventions in PwCRC. Wan et al. (60) investigated the effectiveness of web-based psychosocial interventions and found positive effects of psychosocial interventions on multiple mental health outcomes, QoL, and self-efficacy.

Four reviews (*n* = 25 RCTs) evaluated the effects of psychosocial interventions on QoL (17, 19, 20, 60). Of these, only 3 meta-analyses found statistically significant improvements in QoL after psychosocial interventions (17, 19, 20). Two reviews (17, 20) reported statistically significant improvement in the short-term QoL after psychosocial interventions. Furthermore, Son et al. (19) analysed the subgroups based on the method of delivery and found that only the face-to-face subgroup had statistically significantly improved QoL (*p* = 0.028). For long-term QoL, Dun et al. (17) found significant improvements after cognitive training (SMD = 0.54, *p* = 0.003); however, no significant effect was observed after combined interventions of cognitive training and social support (SMD = 0.50, *p* = 0.435). Zhang et al. (20) found no significant impact on long-term QOL after psychoeducation interventions. Similarly, Wan et al. (60) reported a non-significant improvement in QoL (MD = 2.83, 95% CI = –0.31 to 5.98, *p* = 0.08). Overall, psychosocial interventions had “moderate-quality” evidence in improving short-term QoL of PwCRC.

Two reviews (*n* = 10 RCTs) found mixed results on self-efficacy (SE) outcomes after psychosocial interventions (20, 60). Zhang et al. (20) found enhanced participants’ short-term SE after psycho-education intervention (SMD = 0.71; 95% CI = 0.41 to1.02, *p* < 0.0000), while the findings of Wan et al. (60) favoured the control group (SMD = 0.93, 95% CI = 0.52 to 1.35, *p* < 0.000). Overall, there was “very low-quality” evidence for psychosocial interventions for improved the short-term SE in PwCRC.

Further, 2 reviews (20, 60) investigated the effects of psychosocial interventions in improving mental health outcomes in PwCRC. Both intervention and control groups reported statistically significant reductions in anxiety and depression symptoms after psychosocial interventions. In addition, Zhang et al. (20) showed a substantial reduction in both short-term and long-term anxiety and depressive symptoms. In summary, psychosocial interventions had “moderate-quality” evidence in improving mental health outcomes in PwCRC.

*Lifestyle and multimodal interventions.* Nine reviews (*n* = 134 RCTs) investigated the effects of lifestyle and multimodal interventions in PwCRC (63–69, 78, 79). Of these, 5 reviews (*n* = 82 RCTs) focused on the effects of multimodal prehabilitation interventions in patients undergoing CRC resection (67–69, 78, 79). Four other reviews studied the effects of lifestyle interventions that involved at least 1 lifestyle change in diet, BMI, physical activity level, smoking, or alcohol consumption (63–66).

Multimodal prehabilitation consisted of various exercise, nutritional, and psychosocial interventions that were delivered in hospital, outpatient, or home-based settings before the primary cancer treatment. All 5 reviews (*n* = 35 RCTs) found statistically significant improvements in preoperative functional capacity such as 6MWT after prehabilitation interventions (67–69, 78, 79). In a Cochrane review (67), there was “moderate-quality” of evidence for prehabilitation for improved 6MWT capacity (MD = 24.91, 95% CI 11.24, 38.57; *p* = 0.0004). Overall, prehabilitation interventions had “high-quality” evidence in improving preoperative functional capacity in PwCRC.

Hospital LOS was evaluated in 4 reviews (*n* = 33 RCTs), which reported no significant improvement in hospital LOS after prehabilitation interventions (68, 69, 78, 79). In summary, there was “very low-quality” evidence for prehabilitation interventions for reduced hospital LOS.

Five reviews (*n* = 45 RCTs) studied the effect of prehabilitation interventions on postoperative complication rates in patients undergoing CRC surgery (67–69, 78, 79). One Cochrane review (67) reported low-certainty evidence and non-clinically significant improvement in postoperative complication rates within 30 days after the operation (RR = 0.95, 95% CI = 0.70 to 1.29; *p* = 0.75). Similar findings were confirmed by both Lau et al. (68) and Falz et al. (69). In contrast, 2 recent reviews showed significantly reduced postoperative complications after multimodal prehabilitation intervention (78, 79). Overall, there was “low-quality” evidence for prehabilitation interventions for reduced postoperative complication rates.

Of the 4 systematic reviews (*n* = 52 RCTs) that investigated the effect of lifestyle interventions in CRC survivors (63–66), 3 reviews (*n* = 38 RCTs) pooled data on the outcomes of QoL, mental health, and anthropometric measures (63, 65, 66). A descriptive review found lifestyle interventions in physical activity and/or diet and weight had positive health benefits including improvements in fatigue, physical activity parameters, and overall QoL (64). Aubrey et al. (63) reported improvements in physical QoL favouring healthy eating dietary intervention groups at 12 months follow-up (MD = 1.97, 95% CI = 0.44 to 3.51, *p* = 0.01). Another review showed that non-pharmacological interventions were associated with a statistically significant and clinically meaningful improvement in the QoL scores during 1–4 months of follow-up (SMD = 0.368, 95% CI = 0.070 to 0.665; *p* = 0.000) (66). Similarly, Zhou et al. (65) found higher QoL scores after lifestyle interventions (WMD = 3.12; 95% CI = 0.24 to 5.99; *p* = 0.034). There was “moderate-quality” evidence that lifestyle interventions improve QoL in CRC survivors.

No significant post-intervention effects of lifestyle interventions were observed in 2 reviews regarding mental health outcomes (65, 66). However, subgroup analysis performed by Meng et al. (66) found significant beneficial effects of lifestyle interventions for reduced anxiety and depression at 5–8 months of follow-up. Overall, there was “low-quality” evidence for lifestyle interventions for reduced anxiety and depression symptoms in CRC survivors.

Anthropometric outcomes such as waist circumference and BMI were assessed in 2 reviews with inconsistent results (63, 65). There was “very low-quality” evidence that lifestyle interventions effectively reduced BMI or waist circumference in CRC survivors.

## SUMMARY OF KEY FINDINGS

### Improved physical fitness and functional capacity

- “High-quality” evidence for unimodal or multimodal prehabilitation (moderate-intensity exercise, nutritional, and psychological support) for improved preoperative functional capacity.
- “Moderate-quality” evidence for either home-based or supervised exercise interventions (aerobic, resistance, or combined) at moderate intensity in improving physical fitness, when supervised, or with > 80% of compliance.
- “Low-quality” evidence for either home-based or supervised exercise intervention for improved anthropometric measures (BMI or body fat percentage).
- “Very low-quality” evidence for lifestyle intervention (at least 1 lifestyle change in improving BMI, diet, PA, smoking, or alcohol consumption) for improved anthropometric measures.

### Reduced CRC- or treatment-related symptoms

- “Moderate-quality” evidence for moderate to high intensity, supervised, and combined exercise interventions in improving CRF.
- “Moderate-quality” evidence for acupuncture and related therapies (EA, MA, acupressure, moxibustion, point application, and laser acupuncture) in improving time to first bowel sounds, first flatus, and first defecation after CRC surgery.
- “Low-quality” evidence for moderate intensity, home-based exercise interventions in improving CRF.
- “Very low-quality” evidence for acupuncture and related therapies in reducing postoperative pain.

### Reduced postoperative complication rates

- “High-quality” evidence for perioperative immunonutrition therapy (including at least 2 of arginine, glutamine, omega-3 PUFA, or nucleotide), pro/prebiotics and PUFA supplement in reducing postoperative infectious complications including surgical site infections.
- “Low-quality” evidence for perioperative immunonutrition therapy and glutamine supplements in reducing non-infectious complications.
- “Low-quality” evidence for unimodal or multimodal prehabilitation (moderate-intensity exercise, nutritional, and psychological support) of more than 3 weeks duration in reducing postoperative complications.

### Shortened hospital LOS

- “Moderate-quality” evidence for perioperative immunonutrition therapy, glutamine, arginine, PUFA, and probiotics/prebiotics supplements in reducing LOS.
- “Low-quality” evidence for MA, EA, and acupressure in reducing hospital LOS.
- “Very low-quality” evidence for unimodal or multimodal prehabilitation in reducing LOS.

### Improved psychological well-being

- “Moderate-quality” evidence for psychosocial interventions (psychoeducation, ACT, CBT, peer support, counselling, stress management) in improving anxiety or depression.
- “Very low-quality” evidence for either home-based or supervised exercise interventions in improving anxiety or depression.
- “Low-quality” evidence for lifestyle interventions in improving anxiety and depression at > 4 months of follow-up.
- “Very low-quality” evidence for psychosocial intervention in improving self-efficacy.

### Improved QoL

- “Moderate-quality” evidence for both home-based and supervised exercise interventions at low to moderate intensity to improve QoL, especially for intervention time < 12 weeks and > 80% of compliance.
- “Moderate-quality” evidence for psychological interventions and social support interventions (face-to-face delivery) in improving QoL.
- “Moderate-quality” evidence for lifestyle interventions in improving QoL.

### Improved overall survival

- “Very low-quality” evidence for reduced postoperative mortality rates with perioperative immunonutrition therapy, omega-3 PUFA supplement, and pro/prebiotics supplement.

### Social participation

- Included studies lacked outcome measures for evaluation of societal participations.

## DISCUSSION

With increasing cancer survivorship rates, rehabilitation plays an integral role in comprehensive patient management to maximize functional outcomes (80). There are multiple unmet needs in PwCRC after acute treatment including ongoing physical impairments and psychological distress that causes functional limitations, participation restriction, and reduced QoL (81). While a number of established guidelines on the acute management of CRC and the survivorship care in PwCRC exist (21–23, 82), they lack specific recommendations for structured rehabilitation programmes tailored to this population. This overview summarizes the best available evidence for the effectiveness of various rehabilitation interventions used in PwCRC from published systematic reviews. The findings suggest that rehabilitation interventions have beneficial effects on physical, psychological, functional, and health-economic outcomes in PwCRC. However, the evidence to support the use of many of these interventions remains suboptimal.

Various exercise interventions (aerobic, resistance, low-intensity stretching, or combined) are found to be beneficial in improving self-reported physical function, global QoL, and reducing the manifestation and severity of CRF in people with cancer (83–85). This review highlights significant heterogeneity in the descriptions of exercise interventions (type, frequency, duration, and intensity etc.), leading to inconsistent results. Notably, there is “moderate-quality” evidence for both home-based and supervised exercise interventions (aerobic, resistance, or combined) at moderate intensity in improving physical fitness and QoL in PwCRC, particularly when the home-based interventions have > 80% of compliance rates. There is “low-quality” evidence for moderate intensity home-based exercise interventions (aerobic, resistance, or combined) in improving CRF and “moderate-quality” evidence for supervised and combined exercise interventions at moderate to high intensity in improving CRF. Despite the demonstrated positive benefits of physical activity in alleviating symptoms of depression and anxiety across various cancer cohorts (80), there is still insufficient evidence for either supervised or home-based exercise interventions addressing these psychological outcomes in PwCRC.

Nutrition plays an integral role in modulating immune response and maintaining the health of the gastrointestinal tract in PwCRC. In particular, immunonutrition therapy (including at least 2 of glutamine, arginine, PUFA, or nucleotides) has gained popularity in the perioperative management of gastrointestinal cancers for improved postoperative outcomes (86–88). In an umbrella review, Slim et al. (89) reported significantly fewer postoperative infectious complications and morbidity after perioperative immunonutrition interventions in patients undergoing visceral surgeries. Consistent with these findings, this overview identified “high-quality” evidence supporting perioperative immunonutrition therapy, pro/prebiotics, and PUFA supplements in reducing postoperative overall infectious complication rates in PwCRC. Although Slim et al. (89) could not draw reliable conclusions on the impact of immunonutrition therapy on hospital LOS in a mixed cohort who underwent abdominal surgeries, this review showed “moderate-quality” evidence supporting the use of glutamine, arginine, PUFA, and probiotics/prebiotics supplements either in isolation or combined in reducing hospital LOS in PwCRC. The evidence for the effect of immunonutrition therapy in reducing mortality in PwCRC remains limited due to the scarcity of reported outcomes in current literature, necessitating further research.

Acupuncture and related therapies (EA, MA, acupressure, moxibustion, point application, and laser acupuncture) are commonly practised CAM interventions in many Asian countries. In a recently published umbrella review, Wang et al. (90) reported beneficial effects of 12 different acupuncture interventions in improving postoperative gastrointestinal function and shortening LOS in gastric cancer and CRC, with quality of evidence ranging from “very low” to “moderate”. These findings are comparable to the findings of this overview, which demonstrated “moderate-quality” evidence supporting acupuncture and related therapies in improving postoperative gastrointestinal function and “low-quality” evidence for reduced LOS in PwCRC. However, caution is required when interpreting these findings due to the high level of heterogeneity in the various acupuncture interventions.

There are high levels of unmet supportive care needs, especially related to psychological and social support in the general cancer population, including PwCRC (91, 92). Despite the recognized importance of addressing these needs, there is relatively limited relevant research on psychosocial interventions. Consistent with the published literature in this area, this overview showed mixed findings in evaluating psychosocial interventions. There was “moderate-quality” evidence for psychosocial interventions (psychoeducation, ACT, CBT, peer support, counselling, stress management) in improving anxiety, depression, and QoL in PwCRC, particularly when the interventions were delivered face-to-face. However, more well-designed studies are required to confirm the effectiveness of psychosocial interventions in PwCRC.

There is growing evidence for prehabilitation in improving preoperative functional capacity and postoperative outcomes for patients undergoing various cancer surgeries (93). McIsaac et al. (93) identified “moderate-certainty” evidence supporting improved functional recovery after prehabilitation (unimodal or multimodal) and “low” to “very low” certainty evidence for its effect on reducing the risk of complications, discharge other than home, and LOS. These findings are consistent with our review, demonstrating “moderate-quality” evidence for unimodal or multimodal prehabilitation in improving preoperative functional capacity, “low-quality” evidence for reduced postoperative complications, and “very low-quality” evidence for reduced LOS. Notably, the Enhanced Recovery After Surgery Society Guideline for perioperative care in elective colorectal surgery currently outlines a weak recommendation for prehabilitation (23). Further research that considers the most optimal patient selection criteria and more details on the prehabilitation interventions is needed to inform clinical practice and recommended standards.

There was significant heterogeneity amongst the included systematic reviews, even in those evaluating similar interventions and outcomes. Most reviews included primary studies with significant variability in settings, intervention type, dose or intensity of therapy, mode of delivery, duration of treatment, and timing of interventions relative to the cancer treatment (before, during, or after treatment). This lack of direct comparison of various therapies or programmes from the same intervention category precluded the identification of the optimal or most effective rehabilitation method for PwCRC. Further, the majority of reviews included PwCRC at various stages of their cancer treatment without specifying details of their CRC diagnosis and treatment regimen. Therefore, analyses of the effectiveness of various rehabilitation interventions in PwCRC according to the phase of cancer treatment were not possible. Further, there was substantial heterogeneity within the primary RCTs in the included reviews, including differences in characteristics of participants, descriptions of control arms, assessment time points/follow-up, and outcome measures used. There were also variable adherence rates to the interventions and lost to follow-up data among participants in the primary studies, which contributed to the significant heterogeneity. Therefore, pooling data for quantitative analysis was not possible and best-evidence synthesis was described using a qualitative approach instead. Many included reviews were rated as being of “low” or “very low” methodological quality. The overall quality of evidence for some interventions and outcomes was downgraded from actual evidence reported within primary reviews due to significant imprecision, inconsistency of findings, and the use of different outcome measures. Therefore, caution is required when interpreting the quality of evidence and the external validity of the findings in this study.

To our knowledge, this is the first overview to summarize the evidence from published systematic reviews and/or meta-analyses regarding the effectiveness of various rehabilitation interventions in adult PwCRC. This approach enables direct comparison of results from multiple reviews, leading to a comprehensive evidence-based summary regarding the current state of knowledge. The WHO ICF framework (25) provides a valuable approach for identifying rehabilitation goals in PwCRC. We envisaged that the findings from this overview would provide useful information in helping formulate rehabilitation programmes for PwCRC towards improving their physical impairments, plus functional and participation outcomes. Moreover, this overview highlights the existing gaps in current research, underscoring the need for future studies to address these limitations.

### Study limitations

Despite a comprehensive review of the literature, there are some methodological limitations. First, this review encompassed literature published only in English in specific health science databases, which would have contributed to limitations in the completeness of retrieved literature, as well as selection and reference bias. However, a comprehensive search strategy using broad search terms was used in most relevant databases and bibliography search of relevant articles and journals, and a grey literature search was performed to identify relevant studies. Second, the accuracy of the assessor’s assessment cannot be guaranteed; however, the selection of primary reviews, methodology, and the quality of evidence were all independently performed by at least 2 authors, and all conflicts were resolved by further group consensus. In addition, the review followed the use of widely validated tools to assess the methodology (AMSTAR-2) and quality of evidence (GRADE) of included studies. The methodology quality of the primary RCTs in the included systematic reviews was not assessed; this was beyond the scope of this review. Evaluation of the extent of overlap of the primary RCTs amongst the multiple reviews evaluating similar interventions was also beyond the scope of this overview. Further, due to the significant heterogeneity amongst the included reviews in terms of intervention and patient characteristics, settings, and outcomes, the effects of various rehabilitation interventions were only qualitatively analysed, limiting the generalizability of our findings. There were limited safety and adverse events data regarding various rehabilitation interventions due to incomplete or missing data in the primary reviews. Many of the included reviews had search dates older than 3 years, potentially omitting recent studies. These limitations underscore the need for cautious interpretation of the findings. Future reviews should aim to address these gaps by including more diverse sources, updating evidence, and incorporating quantitative syntheses where possible.

### Conclusions

Rehabilitation interventions play a crucial role in the management and care of PwCRC. The findings demonstrate the beneficial effects of various exercise, nutritional, psychological, acupuncture, psychosocial, lifestyle, and multimodal rehabilitation interventions in improving physical, psychological, functional, societal, and health-economics outcomes in PwCRC. Overall, there remains a lack of high-quality evidence for the effectiveness of many rehabilitation modalities in the CRC population. More robust research evidence for rehabilitation-specific interventions in diverse clinical settings of CRC care is critically needed. Future studies should also encompass a broader range of outcomes, including functional independence, social participation, therapy adherence, adverse effects, and longer-term outcomes (such as survival, mortality) and cost-effectiveness. Furthermore, the adoption of standardized outcome measures will enhance the comparability and the reliability of findings. Addressing these gaps will enhance the development of evidence-based, structured rehabilitation programmes to optimize the needs of PwCRC.

## Supplementary Material


