# Supplementary material for: EFFECTIVENESS OF REHABILITATION INTERVENTIONS IN PATIENTS WITH COLORECTAL CANCER: AN OVERVIEW OF SYSTEMATIC REVIEWS
Source: J Rehabil Med. 2025 Jan 24;57:40021. doi: 10.2340/jrm.v57.40021 (PMC11780671; doi:10.2340/jrm.v57.40021)
Supplement: Supplementary file 1 [file JRM-57-40021-s1.pdf]

Supplementary material has been published as submitted. It has not been copyedited, or typeset by Journal of Rehabilitation Medicine

Appendix S1

Database: Ovid MEDLINE(R) <1946 to May 25, 2023>

Search Strategy:

1 exp Colorectal Neoplasms/ (235999)

2 Colorectal Neoplasms/su (15360)

3 ((colorectal or colon* or colitis-associated or sigmoid or rectal or rectum or anus or anal or colo-rectal or recto-colonic or rectocolonic or rectosigmoid or recto-sigmoid) adj3 (neoplasm* or cancer* or carcino* or tuno* or malign* or adenocarcino* or adenoma* or sarcom*)).tw,kf. (268738)

4 or/1-3 (323339)

5 exp Rehabilitation/ (351354)

6 Rehabilitation Nursing/ (1493)

7 rehabilit*.tw,kf. (219469)

8 prehabili*.tw,kf. (1418)

9 Preoperative Care/ (65288)

10 "Recovery of Function"/ (59093)

11 (non-pharmacolog* or nonpharmacolog* or noninvasive or non-invasive).tw,kf. (245446)

12 rh.fs. (208139)

13 Ambulatory Care/ (46278)

14 ((tele-rehab* or telerehab* or e-rehab* or rehab*) adj3 (remote or virtual or electronic)).tw,kf. (1014)

15 Self Help Devices/ (5670)

16 (virtual reality or VR).tw,kf. (22545)

17 physiotherap*.tw,kf. (34141)

18 ((occupational or physio or physical) adj3 (therap* or treat*)).tw,kf. (59990)

19 exp Physical Therapy Modalities/ (177679)

20 physical activit*.tw,kf. (151437)

21 exp Physical Fitness/ (36167)

22 fitness.tw,kf. (96498)

23 (walk* or walking).tw,kf. (146399)

24 pedomet*.tw,kf. (3168)

25 ((occupational or vocational) adj3 (retrain* or rehab*)).tw,kf. (4381)

26 ((pre-operat* or preoperat* or post-operat* or postoperat* or post-treat*) adj3 (conditioning or exerci* or rehab* or intervention*)).tw,kf. (11118)

27 exp Exercise/ (244930)

28 ((isotonic or isometric) adj3 exerci*).tw,kf. (3407)

29 stretch*.tw,kf. (88175)

30 (exercise or kinesiotherap*).tw,kf. (320482)

31 (strength* or (weight* adj3 (bear* or lift*))).tw,kf. (560604)

32 ((endurance or resistance) adj3 train*).tw,kf. (22014)

33 ((toilet or bowel or bladder or continence) adj3 train*).tw,kf. (1564)

34 exp Home Care Services/ (50776)

35 (homecare or (home adj3 (care or nursing or service* or treat*))).tw,kf. (69737)

36 Dietary Services/ (1419)

37 Dietetics/ (8228)

38 exp Nutrition Therapy/ (113364)

39 dh.fs. (55318)

40 (diet* or food* or nutrit*).tw,kf. (1386403)

41 Dietary Supplements/ (73485)

42 ((dietary or nutrit* or food or herbal) adj3 supplement*).tw,kf. (55171)

43 (polyunsaturated fatty acids or vit* D).tw,kf. (103076)

44 (antioxidant* or anti-oxidant* or folic acid).tw,kf. (308286)

45 n?utr#ceutical*.tw,kf. (12581)

46 Food, Fortified/ (10089)

47 (food* adj3 (enriched or fortified)).tw,kf. (2868)

48 exp Counseling/ (48753)

49 (peer support* or counsel*).tw,kf. (141859)

50 exp Health Education/ (262125)

51 Smoking Cessation/ (32607)

52 (smok* adj3 (quit* or stop* or cessation or ceas* or giv*)).tw,kf. (41739)

53 exp Social Work/ (18604)

54 social work*.tw,kf. (18466)

55 exp Psychotherapy/ (217762)

56 psychotherap*.tw,kf. (53586)

57 ((cognit* or accept* or commit* or behav* or exposure or relaxation or music or art) adj3 (therap* or train*)).tw,kf. (96179)

58 CBT.tw,kf. (14741)

59 (mental adj practice).tw,kf. (436)

60 distract*.tw,kf. (36376)

61 imagery.tw,kf. (19429)

62 mindful*.tw,kf. (16166)

63 hypno*.tw,kf. (26606)

64 (psycholog* adj3 intervent*).tw,kf. (12287)

65 Psychology/ (24120)

66 Psychology, Clinical/ (3270)

67 px.fs. (1179644)

68 (sex* health adj3 (counsel* or educat*)).tw,kf. (1054)

69 exp Electric Stimulation Therapy/ (92566)

70 exp Magnetic Field Therapy/ (16060)

71 ((nerve or electric or transcranial) adj3 stimulat*).tw,kf. (65641)

72 vibration.tw,kf. (36069)

73 (vibro-massag* or (vibrat* adj3 (massag* or therap*))).tw,kf. (501)

74 massag*.tw,kf. (12789)

75 aromatherap*.tw,kf. (1652)

76 exp Acupuncture Therapy/ (28949)

77 (acupuncture or acupressure).tw,kf. (28031)

78 Integrative Medicine/ (1884)

79 exp "Delivery of Health Care, Integrated"/ (14421)

80 (multimodal* or multi-modal*).tw,kf. (74532)

81 ((integrated or holistic or collaborative) adj3 (care or healthcare or health care or medicine or team)).tw,kf. (29334)

82 (multidisciplinary or multi-disciplinary or integrated or interdisciplinary or inter-disciplinary).tw,kf. (483726)

83 ((interdisciplinary or multidisciplinary or multiprofessional or multi-professional or patient) adj3 team*).tw,kf. (42946)

84 exp Patient Care Team/ (72735)

85 or/5-84 (5917472)

86 4 and 85 (45148)

87 review.pt. (3156273)

88 (medline or medlars or embase or pubmed or cochrane).tw,sh. (337486)

89 (scisearch or psychinfo or psycinfo).tw,sh. (58093)

90 (psychlit or psyclit).tw,sh. (918)

91 cinahl.tw,sh. (44067)

92 ((hand adj2 search$) or (manual$ adj2 search$)).tw,sh. (16772)

93 (electronic database$ or bibliographic database$ or computeri?ed database$ or online database$).tw,sh. (57571)

94 (pooling or pooled or mantel haenszel).tw,sh. (145431)

95 (peto or dersimonian or der simonian or fixed effect).tw,sh. (10598)

96 (retraction of publication or retracted publication).pt. (27972)

97 or/88-96 (516456)

98 87 and 97 (219590)

99 meta-analysis.pt. (181346)

100 meta-analysis.sh. (181346)

101 (meta-analys$ or meta analys$ or metaanalys$).tw,sh. (299620)

102 (systematic$ adj5 review$).tw,sh. (325798)

103 (systematic$ adj5 overview$).tw,sh. (3571)

104 (quantitativ$ adj5 review$).tw,sh. (10498)

105 (quantitativ$ adj5 overview$).tw,sh. (423)

106 (quantitativ$ adj5 synthesis$).tw,sh. (4495)

107 (methodologic$ adj5 review$).tw,sh. (8544)

108 (methodologic$ adj5 overview$).tw,sh. (561)

109 (integrative research review$ or research integration).tw. (173)

110 or/99-109 (480311)

111 98 or 110 (564254)

112 86 and 111 (2177)
